# Measuring the Affinities of RNA and DNA Aptamers with DNA Origami-Based Chiral Plasmonic Probes

**DOI:** 10.1021/acs.analchem.2c04034

**Published:** 2022-12-08

**Authors:** Yike Huang, Joonas Ryssy, Minh-Kha Nguyen, Jacky Loo, Susanna Hällsten, Anton Kuzyk

**Affiliations:** † Department of Neuroscience and Biomedical Engineering, School of Science, Aalto University, FI-00076Aalto, Finland; ‡ Faculty of Chemical Engineering, Ho Chi Minh City University of Technology (HCMUT), 268 Ly Thuong Kiet St., Dist. 10, Ho Chi Minh City700000, Vietnam; § Vietnam National University Ho Chi Minh City, Linh Trung Ward, Thu Duc Dist., Ho Chi Minh City700000, Vietnam

## Abstract

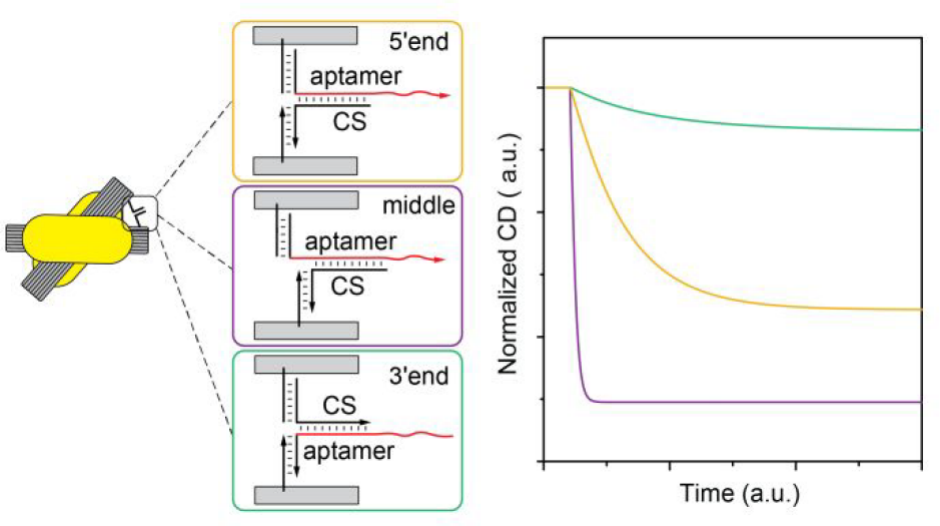

Reliable characterization of binding affinities is crucial for selected aptamers. However, the limited repertoire of universal approaches to obtain the dissociation constant (*K*_D_) values often hinders the further development of aptamer-based applications. Herein, we present a competitive hybridization-based strategy to characterize aptamers using DNA origami-based chiral plasmonic assemblies as optical reporters. We incorporated aptamers and partial complementary strands blocking different regions of the aptamers into the reporters and measured the kinetic behaviors of the target binding to gain insights on aptamers’ functional domains. We introduced a reference analyte and developed a thermodynamic model to obtain a relative dissociation constant of an aptamer–target pair. With this approach, we characterized RNA and DNA aptamers binding to small molecules with low and high affinities.

## Introduction

Aptamers have emerged as promising target-binding ligands with great potential in various application areas. Recently, the combination of aptamers and nucleic acid nanotechnology has opened novel routes for realization of nucleic acid nanostructures with functionalities tailored for bioimaging,^1,2^ biosensing,^3−6^ targeted drug delivery,^7,8^ and therapeutics.^9^ Although over a thousand aptamers have been isolated and reported in the literature, only a few well-characterized aptamer–target pairs have been utilized in the majority of further application developments.^10^ The limited availability of universal approaches to the aptamers’ characterization, especially for small molecule aptamers, is a critical hindrance.^11,12^ For a selected aptamer, the binding affinity to a specific target, which is usually represented by the dissociation constant (*K*_D_), is the key parameter that determines the suitability of the aptamer for specific applications.^13,14^ To measure *K*_D_, differentiating the bound and unbound populations of aptamers is crucial yet difficult.^15^ Currently, the differentiation mostly relies on a particular characteristic of the target–aptamer pair (e.g., changes of size, charge, fluorescence), and the versatility of aptamer targets complicates the partition.^14,16,17^ Labeling the aptamer/target with a fluorophore (e.g., in microscale thermophoresis) or immobilizing the aptamer/target to a surface (e.g., in surface plasmon resonance) is used to ease the difficulty but labeling and immobilization are reported to alter the binding thermodynamics and kinetics.^18,19^ Isothermal titration calorimetry (ITC), which measures the heat of the reaction, is, in principle, widely applicable. However, ITC requires a significant amount of materials that restrict its application.^20^

The competitive hybridization-based approach for measuring *K*_D_ is an alternative universal strategy. Instead of partitioning the aptamer–target complex, the approach discriminates the very distinct states of single-stranded and double-stranded nucleic acids.^21^ The success of the competitive hybridization-based strategy lies in identifying the proper complementary strand because the aptamer rarely interacts with the target as a homogeneous molecule.^22^ The target-induced separation of the aptamer–complementary strand proceeds differently when the complementary strand blocks different regions of the aptamer.^23^ To describe the heterogeneous nature of the aptamer sequence, the abstraction of “domain” is introduced as a consecutive stretch of nucleotides acting as a unit in binding. An aptamer may contain two general domains (essential and nonessential domains) where the essential domain is crucial for target interaction and the nonessential domain contains the sequence that neither interacts with the target nor supports the structural folding. The nonessential domain is to be removed during truncation.^24^ The essential domain can be further divided into noncritical and critical domains. We define the noncritical domain as the sequence that supports the structural folding and the critical domain as the sequence that directly interacts with the target. Complementary strands hybridizing to the nonessential domain of the aptamer can result in the formation of a three-molecule (target-aptamer-complementary strand) side product, which fails to change the hybridized states into the separated states. Complementary strands blocking the critical domain of the aptamer may suffer the risk of forming kinetic traps that hinder the equilibrium of the target-induced separation of the aptamer–complementary strand. The ideal candidates for competitive hybridization-based approaches are complementary strands that bind to the noncritical essential domain of aptamers.

Previously, we developed a competitive hybridization-based approach for characterizing DNA aptamers and provided a thermodynamic model, in which the Gibbs free energies of the hybridization between partial complementary strands and the aptamer were varied to generate various equilibrium states, and the coefficient of determination was used to gain insights into the aptamer domain and to validate the obtained *K*_D_ values.^25^ This method, however, relies on the accurate estimation of the Gibbs free energy of the aptamer–complementary strand hybridization. For DNA–DNA hybridization, the Gibbs free energy can be obtained by the computational tools, such as mfold^26^ or NUPACK.^27^ However, such computational tools are not suitable for estimating the free energies of DNA–RNA hybrids, artificial nucleic acids, and sequences containing complex motifs (e.g., pseudoknots, G-quadruplex). Also, a rather tedious calibration to convert the theoretical free energy to the real free energy in the experimental system was required. Another critical limitation was RNA denaturation during the fabrication of DNA origami-based chiral plasmonic probes.

**Figure 1:**
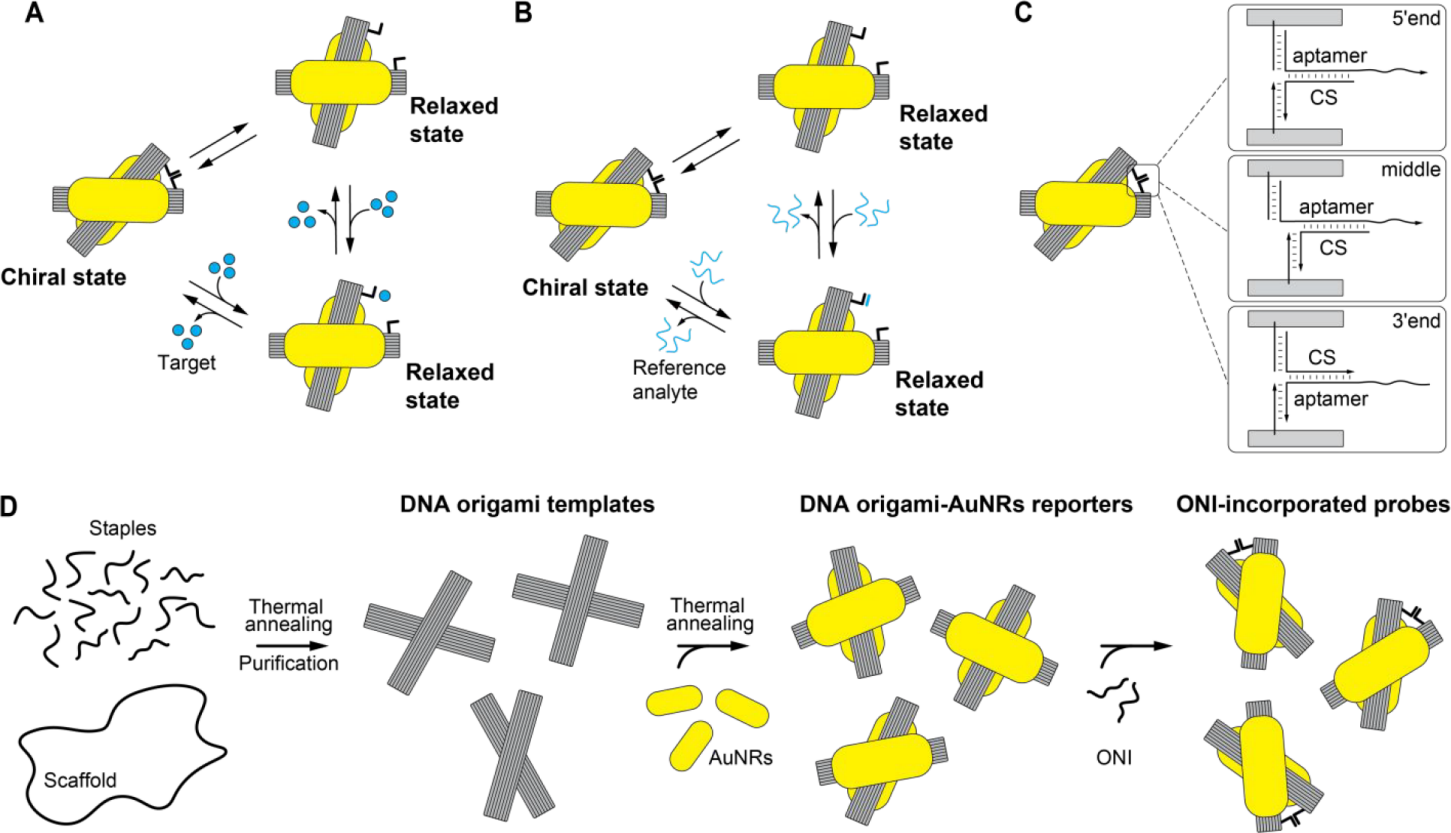
Schematics of ONI-incorporated DNA origami-AuNR probes for measuring aptamer affinities. (A, B) The probes’ states in the presence and absence of the target (A) and the reference analyte (B). (C) The complementary strands hybridizing to different regions of the aptamer. (D) The fabrication process of the probe incorporated with ONI.

To generalize the approach and to expand its applicability to RNA aptamers, here we introduce concepts of the “relative *K*_D_ of aptamer–target” and the “reference analyte”. The reference analyte, which fulfills the criteria of easy accessibility and low batch-to-batch variability (e.g., a competing DNA/RNA strand that displaces the complementary strand) shifts the equilibrium of the aptamer–complementary strand to the separated state just as the target (Figure 1A,B) and serves as a reference point for the relative *K*_D_ of the aptamer–target.^28^ We propose a thermodynamic model that allows obtaining the relative *K*_D_ as a multiplier of the dissociation constant of the aptamer–reference analyte. The absolute *K*_D_ is readily obtained by quantifying the dissociation constant of the aptamer–reference analyte. Importantly, we conduct kinetic measurements for the target-induced separation of different partial complementary strands from the aptamer to screen the proper blocking region to avoid kinetic traps and side products (Figure 1C).

For the optical measurements, we use chiral plasmonic reporters^29−31^ comprised of a reconfigurable DNA origami template and two gold nanorods (AuNRs) to observe the hybridized and separated states of the aptamer–complementary strand. The hybridization and separation of the aptamer and the complementary strand correspond to the chiral state and relaxed state of the probe configuration, respectively (Figure 1A,B). The probes generate strong circular dichroism (CD) signals at the chiral state and weak CD signals at the relaxed state. The high CD signal and the high signal-to-noise ratio of the probe allows for the reliable characterization of the equilibrium populations of the hybridized and separated states of the aptamer and the complementary strand.^25^ Moreover, to maximize the yield and to minimize the possible denaturation of RNA during high temperature annealing in a high salt condition, we incorporated the oligonucleotides of interest (ONI), which are comprised of a pair of aptamer and complementary strand, into the assembled DNA origami-AuNR reporter (Figure 1D). With this fabrication method, the DNA origami-AuNR reporters can be easily turned into chiral plasmonic probes functionalized with a wide selection of aptamers.

## Experimental Section

### Chemicals and Materials

DNA scaffold strands (p 7560) were purchased from Tilibit Nanosystems; core staple strands (SI,Table S1) are from ThermoFisher; thiol-modified DNA strands are from Biomers; other DNA strands are from IDT. Buffers and chemicals were purchased from Fisher Scientific or Sigma-Aldrich unless specified. All reagents were commercially available and used without any further purification.

### DNA Origami-AuNRs Assembly

Scaffold strand (10 nM) and staples were mixed at a 1:10 ratio, and DNA origami was assembled using the established protocol by thermal annealing.^31,32^ For the one-step method, the oligonucleotides of interest (ONI) were added into the staple mixtures. The DNA origami solution was then purified with spin filters for three times. The AuNRs were synthesized using the previously published protocol,^32^ functionalized with 5′-thiol-TTTTTT TTTTTT T-3′ DNA strands by the freeze–thaw method^33^ with DNA to AuNRs ratio of 10000:1, and washed by centrifugation. The DNA origami and AuNR-DNA were mixed at a 1:15 ratio and annealed. The DNA origami-AuNRs with free AuNR-DNA was either directly used for the next step or purified through gel electrophoresis. Details of the methods are described in the SI.

### Incorporation of Oligonucleotides of Interest (ONI)

For the two-step method, the DNA origami-AuNRs were mixed with the ONI in TBE buffer containing MgCl_2_ and NaCl (SI, Table S2). The ratio of ONI and DNA origami-AuNRs was varied between 2:1 and 250:1. The samples were either incubated at room temperature overnight or annealed from 42 to 20 °C.

### Circular Dichroism (CD) Measurements

For kinetic experiments, 7 μL solutions of the ONI-DNA origami-AuNRs probes were added to the 63 μL reaction buffer containing reference analyte or target and mixed by vortex. The 70 μL samples were immediately pipetted into the cuvette and the CD amplitude at 620 nm was recorded with the Jasco J-1500 CD spectrometer. The CD amplitude of the aliquot ONI-DNA origami-AuNRs sample that underwent the same operation in the reaction buffer without target or reference analyte was used as the control points before target binding. For thermodynamic experiments, the samples of ONI-DNA origami-AuNRs were incubated in 70 μL reaction buffer containing different concentration of reference analyte or target at room temperature with shaking at 100 rpm. The CD spectra were measured after overnight incubation.

## Results and Discussion

### Incorporation of Oligonucleotides of Interest

Previously, ONI-like sequences were often incorporated during the DNA origami assembly (one-step method),^31,3,25^ followed by the AuNRs attachment (SI, Figure S1A). Here, we altered the fabrication process by inserting the ONI directly into the assembled DNA origami-AuNRs reporters (two-step method; SI, Figure S1B). To incorporate the ONI, which comprises an aptamer and a complementary strand (CS), into the DNA origami-AuNRs reporter, tail sequences (13 nt) were added to the end of the ONI sequences (ONI-tail) for hybridizing with the docking sequences on the DNA origami (SI, Figure S2 and Table S3). We used three representative sequences (Apt1, Apt1R, and Apt2) of 15 nt and the corresponding partially complementary strands (CS1 (10 nt), CSR1(9 nt), and CS2 (10 nt)) to investigate the incorporation (sequences in SI, Table S4). All the sequences were predicted to have zero free energy by themselves using NUPACK.^27^ The hybridization lengths of the demonstrative DNA–DNA pairs (ONI-1 comprised of Apt1-CS1 and ONI-2 comprised of Apt2-CS2) and the RNA–DNA hybrid pair (Apt1R-CSR1) were fixed at 10 or 9 bp, respectively. When the tail sequences were added to Apt and CS, the sequences of ONI-1-tail (Apt1-tail and CS1-tail) remain relatively linear (the Gibbs free energy of secondary structure > −3 kcal/mol), while the ONI-2-tail (Apt2-tail and CS2-tail) forms relatively stable hairpin structures (the Gibbs free energy < −3 kcal/mol;^34^ see details in SI, Figure S3 and Table S4).

**Figure 2:**
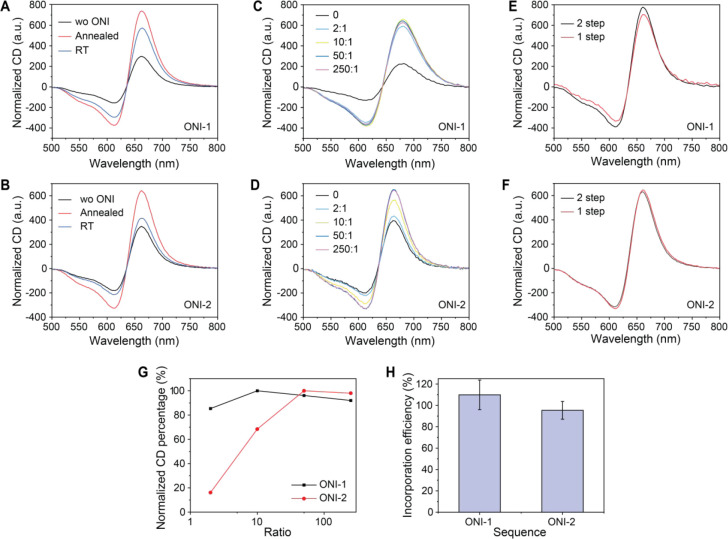
Incorporation of ONI into the DNA origami-AuNRs reporters. (A, B) The CD spectra of the probes after incubating the reporter with ONI-1-tail (A) and ONI-2-tail (B) strands (ONI-tail to reporter ratio 50:1) with annealing treatment and room temperature (RT) incubation. The CD amplitude is normalized by dividing with the absorption value at 650 nm. (C, D) The CD spectra of the probes after incubating the reporters with ONI-1-tail (C) and ONI-2-tail (D) at different concentration ratios with annealing treatment. (E, F) The representative CD spectra of the probes incorporated with ONI-1 (E) and ONI-2 (F) using the two-step method at the optimized condition and the one-step method. (G) Comparison of the trend of the incorporation efficiency with ratio for ONI-1 and ONI-2. The normalized CD signals at 620 nm were used for calculating the percentage. The value at the optimized condition was set as 100%. (H) The incorporation efficiency of the two-step method compared to the one-step method.

We first explored the influence of temperature on ONI incorporation by comparing the thermal annealing and the room-temperature incubation. As shown in the Figures 2A,B, the CD signals increased after incubating the reporters with ONI-tail strands in both treatments. The higher CD signal of annealed samples indicated an improved incorporation efficiency compared to room temperature incubation, especially for the ONI-2-tail, which requires additional energies to disrupt the intramolecular base pairs in the secondary structures to allow the intermolecular hybridization to happen. The TEM images demonstrated similar integrities of the DNA origami-AuNRs probes after the two treatments (SI, Figure S4A,B). Also, when the reporters were annealed without ONI, the CD signal remained the same (SI, Figure S4C), indicating that the increase of the CD signal originated from the incorporation of the ONI instead of temperature effects on the quality of the reporters.

Then, we explored the effects of the concentration ratio between the ONI-tail and the DNA origami-AuNRs reporters. Different behaviors were observed: for the ONI-1-tail, the CD reached its maximum at a ratio of 10:1 and slightly dropped with a further increase of the ratio (Figure 2C,G); for ONI-2-tail, the CD reached its maximum at the ratio of 50:1 (Figure 2D,G). Finally, we calculated the incorporation efficiency of the two-step method at the optimized condition using the samples with an ONI-tail inserted during the DNA origami folding (one-step method) as the reference of complete integration. The Figure 2E,F showed the typical CD spectra of the probes prepared by two methods. During the preparation of the ONI-DNA origami-AuNRs probes, operational errors can accumulate at the steps of the folding and purification of DNA origami, DNA functionalization of AuNRs, and the assembly and purification of the DNA origami-AuNRs constructs. Consequently, the absolute CD signal of the final ONI-DNA origami-AuNRs product can vary. To compare ONI-DNA origami-AuNRs probes fabricated with different workflows, three independent experiments were conducted. Both pairs of ONI demonstrated high incorporation efficiencies in the two-step method, with yields comparable to the one-step method (Figure 2H).

Finally, we substituted the DNA/DNA ONI with the hybrid of RNA/DNA (Apt1R-tail/CSR1-tail) and observed similar trends of the temperature and ratio effects (SI, Figure S5). Also, the incorporation of Apt1-tail/CS1-tail for unpurified reporters (with free DNA-functionalized AuNRs suspended in the solution) performed the same as the purified reporters (SI, Figure S6).

### Design of the Reference Analytes

Short DNA and RNA are commonly available molecules with low batch-to-batch variability across manufacturers. In addition, the sequences of DNA and RNA can be rationally designed as competing strands to hybridize with aptamers for displacing the complementary strand^35,36^ and, thus, to serve as reference analytes. We propose that the competing strand shares a similar length to the complementary strand so that the *K*_D_ of aptamer-competing strand is low enough to shift the equilibrium of aptamer-complementary strand but high enough to exhibit a concentration dependency behavior in a reasonable concentration range. The toehold length of the competing strand is long enough to render fast reactions^37,38^ and short enough to guarantee sufficient overlapping so the aptamer-complementary strand is fully dissociated upon competing strand binding, avoiding the formation of the complex of competing strand-aptamer-complementary strand.^39^

**Figure 3:**
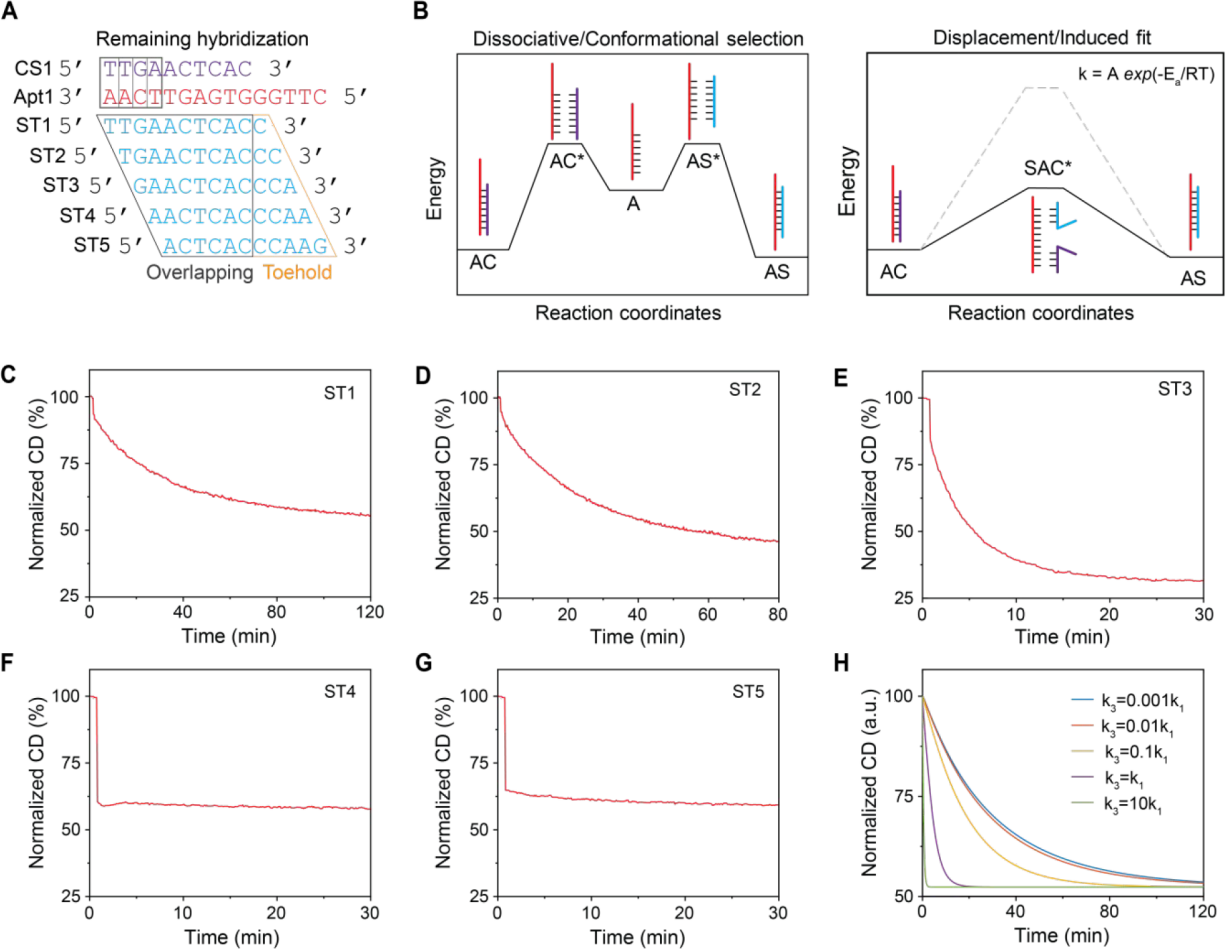
Kinetics of the reference analyte induced separation of aptamer-complementary strand. (A) The sequence of the Apt1, CS1, and the competing strands with different toehold, overlapping, and remaining hybridization lengths. (B) The energy landscapes of the dissociative (conformational selection) pathway and the displacement (induced fit pathway). (C–G) The normalized CD amplitude, indicating the hybridized state of Apt1-CS1, after the addition of different competing strands at time point around 1.5 min. (H) The simulation of the reaction kinetics of competing strands with different toehold lengths and, consequently, different displacement reaction rate constants.

To understand the kinetics of the strand displacement in our system with short (9–10 bp) hybridization, we incorporated the ONI-1 in the DNA origami-AuNRs reporter and tested five different DNA competing strands (ST(1–5)) as potential reference analytes (Figure 3A). All five ST strands form 11 base pairs with the Apt1 but with different toehold lengths and consequently different reaction energy landscapes (Figure 3B). The overlapping length, counting the base pairs shared by the two duplexes of Apt1-CS1 and Apt1-ST, equals to the total length of ST (11 nt) minus the toehold length. The remaining hybridization lengths between Apt1 and CS1 after binding with ST correspond to the total length of CS1 (10 nt) subtracting the overlapping lengths. Usually, the double helix under 5 bp is unstable. Thus, we set the toehold lengths of the reference analytes from 1 to 5 so that the remaining hybridization lengths between Apt1 and CS1 are 0–4 bp to ensure the full separation of Apt1-CS1 upon ST binding. We tested the kinetics of the strand displacement with different competing strands (1.1 μM) at room temperature and concluded that 1–2 nt toehold allowed the equilibrium to be reached within hours (Figure 3C,D). The competing strand with 3 nt toehold rendered an equilibrium time within 30 min (Figure 3E). The toeholds of 4–5 nt enabled the completion of the reaction within 1 min (Figure 3F,G). Therefore, we consider 4 nt as a sufficient toehold length for a reference analyte in our system.

To explain the different kinetic behaviors, we conducted a semiquantitative simulation in MATLAB (code in the SI). Three reactions are involved:
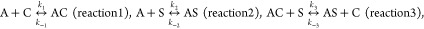
where A, C, and S stand for the aptamer, the complementary strand, and the reference analyte, respectively. In this specific case, A, C, and S represent Apt1, CS1, and ST. Two pathways are expected to be included:^40^ (i) dissociative (conformational selection) pathway: AC duplex first separates to A and C and then A binds with S (reactions 1 and 2, Figure 3B); (ii) displacement (induced fit) pathway, which can be simplified as AC interacts with S to form a transient intermediate SAC* complex and then collapses to AS and C (reaction 3, Figure 3B). Since reactions 1 and 2 are hybridization reactions of two linear strands that have no repetitive sequences and share similar lengths and GC contents, we assume their forward reaction rate constants are the same (*k*_1_ = *k*_2_).^41−43^ As shown in the Figure 3H, when the forward rate constant of the displacement reaction (*k*_3_) is much smaller than the forward rate constants of the hybridization reaction (*k*_3_ ≪ *k*_1_ = *k*_2_), the dissociative pathway dominates and slowly reaches equilibrium; when the three rate constants are equal or *k*_3_ is larger than *k*_1_ and *k*_2_, the reaction is dominated by the displacement pathway with a fast saturation. The results correspond to the kinetics for the five competing strands. When the toehold is short, due to the high active energy (*E*_a_) to form the transient state SAC*, *k*_3_ is small and the kinetic is slow; on the other hand, when the toehold length is long, the active energy to form the transient state SAC* is lowered to a similar level as AC* and AS*, producing a large *k*_3_ comparable to *k*_1_ and *k*_2_ and the kinetic is fast.

### Screening Complementary Strands for Aptamers

Considering the heterogeneous nature of the functional domains of aptamers when interacting with the target, a set of partial complementary strands, which hybridize with different regions of the aptamer, were explored. The complementary strands share a fixed length (9 or 10 nt), which is short enough to allow a relative fast dissociation in the presence of the target but long enough for the equilibrium to favor the hybrid aptamer-complementary strand state in the absence of the target to generate a strong CD signal at the initial state.

**Figure 4:**
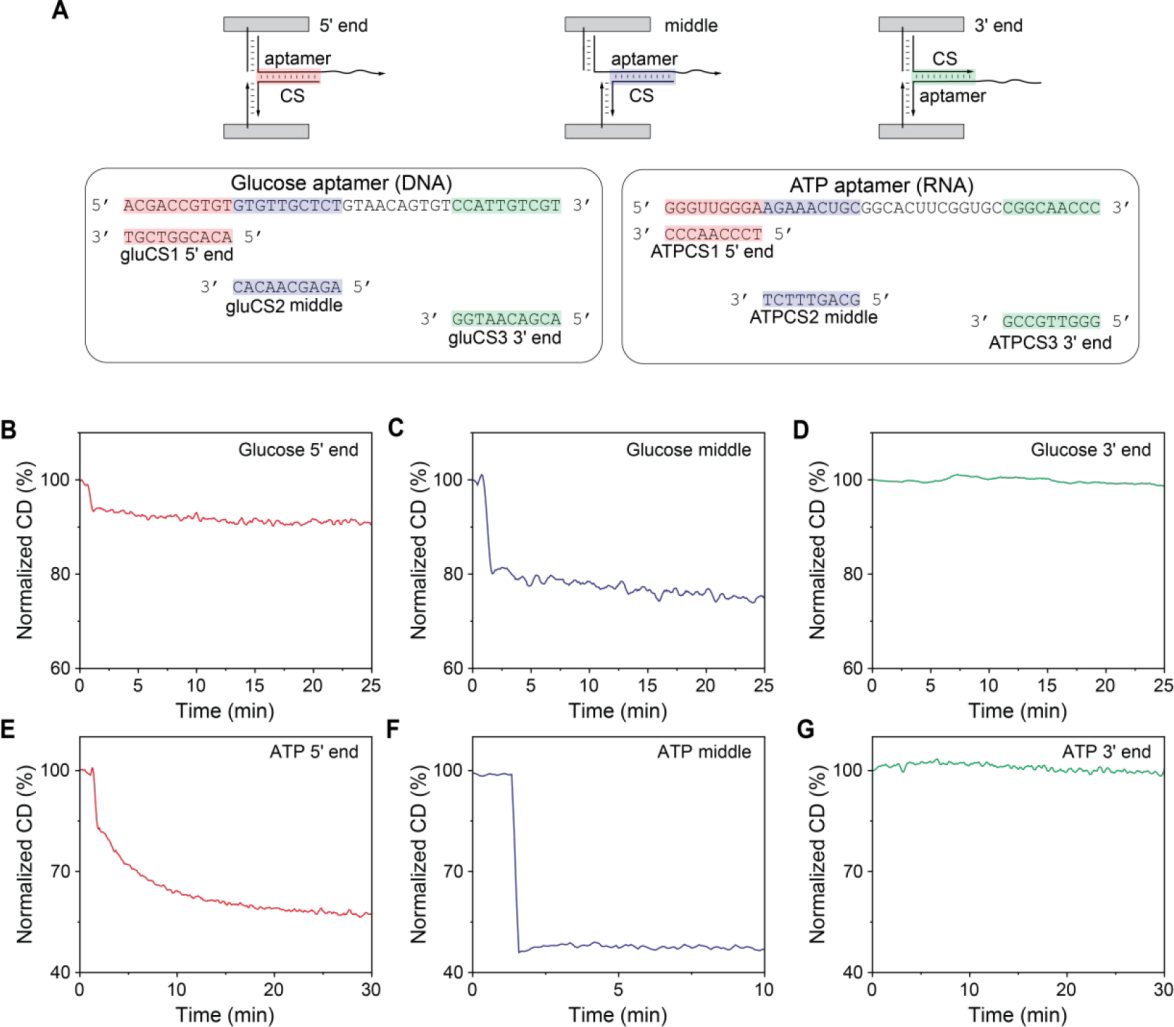
Kinetics of the target-induced separation of aptamers and complementary strands. (A) The schematics of the complementary strands hybridizing to the 5′ end (left), the middle (middle), and the 3′ end (right) regions of the aptamer in the probe and the sequences of the aptamer and the complementary strands. (B–G) The normalized CD amplitude percentage compared to the initial state (100%), which indicates the hybridization states of the glucose aptamer and the complementary strands hybridizing to the 5′ end (B), the middle (C), and the 3′ end (D) regions, after the addition of glucose at time point around 1.5 min. (E–G) The normalized CD amplitude, indicating the hybridization state of the ATP aptamer and the complementary strands hybridizing to the 5′ end (E), the middle (F), and the 3′ end (G) regions, after the addition of ATP at time point around 1.5 min.

We first used a DNA glucose aptamer (40 nt)^44^ as an example to show that the choice of complementary region significantly affected the target binding-induced separation of aptamer-complementary strand (Figure 4A and SI, Table S5). Tail sequences were added to the 3′ end or the 5′ end of the glucose aptamer without disruption of the secondary structure of the aptamer (SI, Figure S7). Three different 10 nt complementary strands (gluCS1, gluCS2, and gluCS3), which hybridize to the 5′ end, the middle region, and the 3′ end of the aptamer sequence, were incorporated into the reporter together with the aptamer (SI, Table S6). We performed kinetic measurements to investigate the separation of the aptamer and the complementary strand induced by the glucose (100 mM). As shown in the Figure 4B–D, for the samples with the complementary strands hybridizing to the 5′ end and the middle region, the CD amplitude reduced immediately after adding glucose. However, the CD signal remained almost the same for the sample with the complementary strand hybridizing to the 3′ end of the aptamer.

Next, we explored the separation kinetics of the RNA ATP aptamer (40 nt)^45,46^ and its partially complementary DNA strands (9 nt) upon target binding (Figure 4A and SI, Figure S8 and Tables S5 and S6). As shown in the Figure 4E–G, the CD signal decreased immediately after adding ATP (100 μM) for the sample with the complementary strands hybridizing to the middle region of the aptamer but exhibited little change for the sample with the complementary strand hybridizing to the 3′ end of the aptamer. When the blocked region was at the 5′ end of the aptamer, the CD changed slower compared to the middle part, but the equilibrium was still reached within 30 min.

The phenomena of different kinetic behaviors of the separation of aptamer-complementary strand pairs induced by the target can be described by the energy landscapes similar to the strand displacement (Figure 3B). Three reactions are involved:
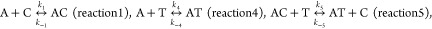
where A, C, and T stand for the aptamer, the complementary strand, and the target, respectively. Depending on the aptamer’s domain blocked by the complementary strand, the energy of the intermediate target-aptamer-complementary strand complex (TAC*) in the induced fit (displacement) pathway may vary significantly. For example, when the complementary strand binds to the nonessential domain of an aptamer, which neither interacts with the target nor supports the structural folding, the TAC* complex is energetically stable enough to persist as a side product; when the complementary strand binds to the noncritical essential domain, the exposed critical domain may serve as a “toehold” and allow the initial interaction with the target, so the TAC* is energetically possible as an intermediate but unstable, enabling a fast separation through the induced fit pathway; when the complementary strand binds to the critical domain, TAC* is energetically unlikely and the induced fit pathway is hindered so only the slow conformational selection (dissociative) pathway is allowed. From the energy landscape point of view, the target-induced separation of aptamer and complementary strands resembles the strand displacement. The structures of real aptamers, however, are often more complex, involving various canonical and noncanonical base interactions. Therefore, the conformational selection pathway can be even slower for aptamers due to extra folding steps. For example, the representative Apt1 with linear structure can directly bind to ST after dissociating from CS1 while real aptamers may require the formation of functional structures (e.g., hairpin, three-way junction, G-quadruplex) before interacting with their targets.

### Calculation of the Relative Dissociation Constant

To measure the affinity of an aptamer (A) to a specific target (T), we introduced the reference analyte (S) and developed a thermodynamic model to calculate the relative dissociation constant of the aptamer-target (AT) binding compared to the dissociation constant of the aptamer-reference analyte (AS) binding without the preknowledge of the dissociation constant of the aptamer-complementary strand (AC).

**Figure 5:**
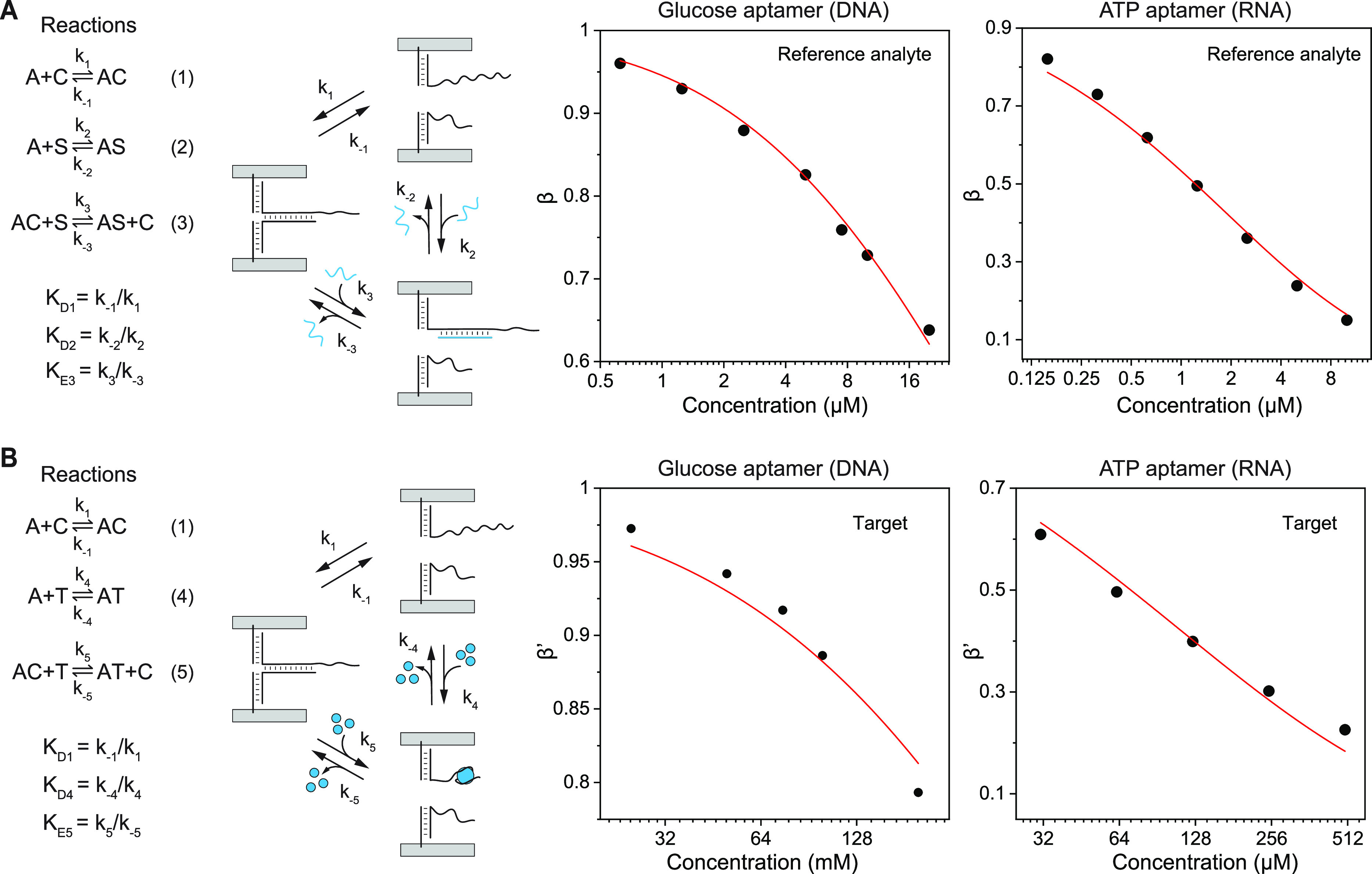
Thermodynamics of the target-induced separation of aptamers and complementary strands. (A) The reactions involved in the equilibrium states in the presence of reference analytes and the dependence of β with the concentration of the reference analytes (gluS or ATPS). (B) The reactions involved in the equilibrium states in the presence of targets and the dependence of β′ with the concentration of the targets (glucose or ATP).

At the equilibrium states, in the samples without the reference analyte or the target, the hybridization and separation of the aptamer and the complementary strand (Figure 5A,B, reaction 1) is at equilibrium; in the samples with the reference analyte, besides reaction 1, the binding of the reference analyte to the free aptamer (Figure 5A, reaction 2), and the displacement of the complementary strand by the reference analyte (Figure 5A, reaction 3) are also at equilibrium; in the samples with the target, the reactions involving the aptamer, the complementary strand, and the target (Figure 5B, reactions 1, 4, and 5) are at equilibrium. We use *K*_D1_, *K*_D2_, and *K*_D4_ to stand for the dissociation constants of the aptamer-complementary strand, the aptamer-reference analyte, and the aptamer-target, respectively; *K*_E3_ and *K*_E5_ are the equilibrium constants of the displacement reactions 3 and 5, with  and . Therefore, the dissociation constant of the aptamer-target (*K*_D4_) equals to the dissociation constant of the aptamer-reference analyte (*K*_D2_) multiplied by the ratio between the equilibrium constants of the displacement reactions (eq 1).
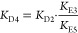
1
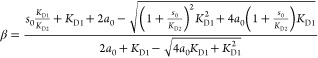
2
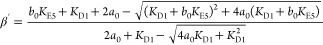
3

To obtain the equilibrium constants of the displacement reactions (*K*_E3_ and *K*_E5_), we defined parameters β as the ratio of the AC concentrations in the presence and absence of the reference analyte; β′ as the ratio of the AC concentrations in the presence and absence of the target.

On one hand, β is a function of the *K*_D1_, *K*_D2_, *a*_0_, and *s*_0_ (eq 2) and β′ is related to the *K*_D1_, *K*_E5_, *a*_0_, and *b*_0_ (eq 3; see deductions in SI, Theoretical Model section). Here, *a*_0_ is the input local concentration of the aptamer and complementary strand which are linked on the same DNA origami. The local concentration, which is mainly determined by the geometry of the nanostructure was roughly estimated as 70 μM by calculating the two molecules confined in a defined volume constrained by the geometrical parameters of the DNA origami (details in SI, Theoretical Model section). This value agrees well with the previous experimental measurement.^25^ The *b*_0_ and the *s*_0_ stand for the input bulk concentrations of the target and the reference analyte, respectively.

On the other hand, β and β’ are the ratios of the relative normalized CD signals of the probes in the presence and absence of the reference analytes and targets, respectively (see deduction in SI, Theoretical Model section). We varied the input concentrations of the reference analyte (*s*_0_), measured the corresponding CD signals of the probes, and calculated the corresponding β. By fitting β with *s*_0_ using eq 2 and setting the *K*_D2_ as a free parameter (SI, Data Analysis section), we obtained the corresponding *K*_D1_ and subsequently *K*_E3_ that equals to . Similarly, after obtaining *K*_D1_, we varied the target concentration (*b*_0_) and measured the corresponding CD signals to calculate the corresponding β′. We gained *K*_E5_, by fitting β′ with *b*_0_ using the eq 3. Then, we obtained , which remained similar in a wide range of manually adjusted *K*_D2_. The SI, Figure S9 describes the whole workflow.

First, we used the DNA glucose aptamer to demonstrate the validity of the model. The complementary strands hybridizing to the 5′ end and the middle region are both potential candidates as the reaction achieved equilibrium in a reasonable time scale according to the kinetic result. However, the probe configuration is optimized when the complementary strands hybridize at the end of the aptamer, generating a stronger CD signal at the initial state (SI, Figure S10). Therefore, for the affinity measurement, we chose the 10 nt complementary strand (gluCS1, 5′-ACACGGTCGT-3′) that hybridizes to the 5′ end of the aptamer. The reference analyte, a 10 nt DNA strand (gluS, 5′-ACACACACGG-3′) hybridizing with the aptamer to displace the complementary strand with a 4 nt toehold, was designated accordingly. The concentration of the input reference analyte (*s*_0_) was varied from 0.625 to 20 μM, and the corresponding CD signals of the samples were measured after overnight incubation to calculate β (SI, Figure S11A). As shown in the Figure 5A, by fitting β with the corresponding *s*_0_, *K*_D1_ was obtained as 1.29 ± 0.0585 μM with *R*^2^ = 0.992 (*K*_D2_ was set as 1 μM). Therefore, *K*_E3_ was calculated as 1.29. In parallel, samples were treated with various concentrations of glucose (*b*_0_: 25–200 mM) and the corresponding β′ values were calculated by measuring the CD signals (SI, Figure S11B). The equilibrium constant *K*_E5_ was obtained as (3.51 ± 0.295) × 10^–5^ with *R*^2^ = 0.955 by fitting β′ with *b*_0_ (Figure 5B). Dividing *K*_E3_ with *K*_E5_ generated the ratio as 3.68 × 10^4^. Therefore, the dissociation constant of the aptamer-glucose (*K*_D4_) was approximately 37000 times the dissociation constant of the aptamer-reference analyte (*K*_D2_). We estimated the *K*_D2_ as 0.139 μM using the system parameter described in the previous study.^25^ The absolute value of the *K*_D4_ was calculated as 5.1 mM, similar to the values reported in the literature.^25,44^

Then, the RNA ATP aptamer was investigated with the same workflow. The complementary strand (ATPCS1, 5′-TCCCAACCC-3′) hybridizing to the 5′ end of the aptamer for 9 bp was chosen. A 10 nt RNA strand (ATPS, 5′-UUUCUUCCCA-3′), which hybridizes with the aptamer and displaces the complementary strand, was designated as the reference analyte. The samples were treated with varied concentration of the reference analyte (*s*_0_: 0.156–10 μM; SI, Figure S12A) and β was fitted with *s*_0_ (Figure 5A). At *K*_D2_ = 0.01 μM, the *K*_D1_ was obtained as 0.353 ± 0.00982 μM with *R*^2^ = 0.998, and thus, *K*_E3_ as 35.3. The samples treated with ATP (*b*_0_: 31.3–500 μM) generated a series of CD signals (SI, Figure S12B). By fitting β′ with *b*_0_, *K*_E5_ was obtained as 0.594 ± 0.0571 (Figure 5B). As the ratio between the equilibrium constants of displacement reactions was 59.5, the affinity of the aptamer to ATP, thus, was approximately 60× lower than to the reference analyte. As *K*_D2_ of RNA–RNA hybridization can be readily obtained by many sensing schemes as well as standard techniques, for example, UV melting,^47,48^ the absolute value of *K*_D4_ can be estimated.

## Conclusions

We provide a strategy to combine the domain investigation and *K*_D_ measurement and to potentially serve as a workflow for designing and testing biosensing schemes. The competitive hybridization-based strategy, in principle, is applicable to most of the aptamers including DNA, RNA, and modified nucleotides such as locked nucleic acid (LNA) and peptide nucleic acid (PNA). The approach, however, may not apply to some aptamers with extensive intramolecular hybridization, as the complementary strand may act as a partial aptamer. To solve the limitation, a split aptamer strategy may be used instead.^49^ Although, the hybridization energies may require calibration for different systems, the *K*_D_ of two oligonucleotides usually can be measured robustly. By introducing the DNA/RNA reference analyte and relative *K*_D_, we provide a solution to address the discrepancy in the reported affinities of aptamers by converting the inconsistent values into a commonly agreed value. We expect that our results may contribute to bridging the gap between upstream-selection and downstream-applications of aptamers and advance the development of nucleic acid nanotechnologies.

## Supporting Information Available

Sequences, equation derivation, data analysis, MATLAB code, and other supporting data (PDF)
