# Supplementary material for: Measuring the Affinities of RNA and DNA Aptamers with DNA Origami-Based Chiral Plasmonic Probes
Source: Anal Chem. 2022 Dec 8;94(50):17577–86. doi: 10.1021/acs.analchem.2c04034 (PMC9773176; doi:10.1021/acs.analchem.2c04034)
Supplement: Supplementary file 1 — ac2c04034_si_001.pdf [file ac2c04034_si_001.pdf]

Supplementary information for:

Measuring the affinities of RNA and DNA aptamers with DNA origami-based chiral plasmonic probes

Yike Huang[1,*], Joonas Ryssy[1], Minh-Kha Nguyen[1,2,3], Jacky Loo[1], Susanna Hällsten[1], Anton Kuzyk[1,*]

[1] Department of Neuroscience and Biomedical Engineering, School of Science, Aalto University, FI-00076 Aalto, Finland

[2] Faculty of Chemical Engineering, Ho Chi Minh City University of Technology (HCMUT), 268 Ly Thuong Kiet St., Dist. 10, Ho Chi Minh City, Vietnam

[3] Vietnam National University Ho Chi Minh City, Linh Trung Ward, Thu Duc Dist., Ho Chi Minh City, Vietnam

* To whom correspondence should be addressed. E-mail: yike.huang@aalto.fi Correspondence may also be addressed to E-mail: anton.kuzyk@aalto.fi

Contents

1. **Materials** 2
2. **Methods** 6
3. **Design benefits** 8
4. **Results** 9
5. **Theoretical model** 14
6. **Data analysis** 17
7. **MATLAB code** 20

1. Materials

DNA scaffold strands (p7560) were purchased from tilibit nanosystems; core staple strands (SI Table S1) from ThermoFisher; thiol modified DNA strands from Biomers; other DNA strands from IDT. Buffers and chemicals were purchased from Fisher Scientific or Sigma-Aldrich unless specified. All reagents were commercially available and used without any further purification. Type I ultrapure deionized (DI) water from the Milli-Q system was used for all experiments. Spin filters for DNA origami purification (cut off size 100 kDa) were manufactured by Millipore.

Table S1. Core staple strands of DNA origami.

Start	End	Sequence (5’-3’)
4[34]	13[34]	AATAATTTCAGCGGCTACGAATACACTA
8[90]	6[77]	AGGTCCGGATATTCTGACGAGGATGGTT
24[146]	18[133]	AGTAATCTTTTAGTCTA GAAAAAGCCTA
25[161]	16[161]	GAAATACCATTGCATTAAGCAGCCTTTA
26[146]	16[133]	TATTAATCAAGGCATAAAAATTTTTAGA
16[104]	14[91]	TCATAGGTCTGAGAAAAACATCACGAAT
22[146]	23[146]	TCTTTAATGCGCAGTTAGAGCCGTAAAA
25[203]	17[216]	GTAGCAAATCGGCCCCATAAATTAATGC
18[34]	27[34]	GGGGTGCCAATTCCAACGTCACCACTAC
13[35]	1[48]	AAACACTTGAGTTTGTTAGCGTAACGAT
4[223]	13[223]	AGCCTAACAATTTTTTCAACCAGGTGGC
25[245]	16[245]	GAGCTAAGCTTTCCGCGATCGGGCGATT
2[139]	0[126]	GCGAGAGCCAGACGCACCCTCAGAACCG
26[62]	16[49]	GATTACAAACAGTACTTCTGTAAATCCC
4[76]	13[76]	CGTTGGGGGAACAATTGTATCGCGCGAA
4[118]	13[118]	TGAATCCCTGCGGATAGCTCATAACAGT
26[230]	16[217]	CTGAGTAGGTAGCTCAGGGTTTTTTCAA
23[203]	18[203]	GGCCTTCAAGAGTCACGGTAACTGGAGC
27[140]	15[153]	CGAACGTATGATACATGCAATGCCTGAG
9[245]	4[238]	ATCGATATAGAGCCGAGGCAGGAATGGAAACTAAC
2[202]	13[195]	TTTAGCGCCGGTATGAGGGTTGATATAATTAGCAA
12[174]	12[182]	TAAGATCAAGCCAACGCTCATAGCCGAAAGAACTG
27[98]	16[105]	CTAAAGGCGGTGGGAGCGATAGCTTAGAATCAAAA
25[140]	22[147]	ATCCTTTGCCCGACCACGACCGGGACATCTATTAG
1[49]	12[42]	CTAAACAATGTACCGTAACACCATCTTTAATACGT
23[175]	19[195]	CCTTCTGACCTGAAAAATGGAAATACCGACCGTAA
18[132]	21[139]	CTAATAGCTTTTTCAGTGCCATATCTGGTCGAACT
6[97]	8[91]	TAAATTGGGCTTGAAAACACCAGAACGACTCCAAC
27[77]	16[84]	GTAAAGCCCTTGAACCTTAGAATCCTTGGACTACC
5[84]	9[90]	CATTAAGAAAACGATTAATCATAAGGGAACAGGAT
4[202]	13[209]	AATAAACTTGCTATACGGAATACGTAGAAAATACA
22[83]	22[91]	AATATACTTTGAATACCAACACCTTGCTTTTAACG
19[196]	23[202]	TCGATGATGTCCATCACGCTGAGTAATTCGCGTCT
2[181]	13[174]	CGGGAGGAATCAGACCGGAATAGGTGTATTCTCCT
6[153]	9[160]	AATAGATATGCAGAACGCGCCTCTGTCCAAGTACC
25[77]	18[84]	CAATATACGGAATTTGAACCTCCGGCTTACATTTA
21[196]	21[188]	TTTTGTTCGCCATCAAAAACGTAAGAATCCTAAAC
1[154]	13[160]	TACCGCGACTCAGGAGGTTTATTAGTTTGACCATT
22[209]	21[195]	ATAGGAAAAAATTCGATTGTATAAGCAAATATTTAGTTAATA
7[175]	7[174]	ACTGAACACAAGAATTGAGTAAAGTAATTGACGGGAGAATTA
19[168]	22[168]	ATTTAATTAAAACATACCGAACGAACCAACGTGGCACAGACA
24[111]	20[98]	GTATTTCATCTTTAGGAATTGAGGAAGGGAACCTCGAAAAAT
27[56]	17[69]	GTTTTTTTCACATACGTCGCTATTAATTAACCTTGCATAAAT

4[139]	11[139]	CTTATCATTCCAAATTACCCTGACTAACCCAACATGCATCAT
22[251]	21[237]	GGATAGGTGCATCTCCAGCTTTCGGTTGATAATCAGCGCATC
7[140]	7[139]	TCAGCTAAAGTCCTGATTATAGTCAGAAATCGCGTTTCATGT
23[77]	20[77]	TTGCGTACCTACCAATGAAACAAACATCATGTTACAAAATCG
22[62]	22[63]	TTACATCGGGAGAGAATCCTGTTTAAAACAGAAATGTACCTT
12[195]	8[182]	ACCCAAACAAAGTTTAAGAAACAAGAAACAGAGAGATAACCC
3[238]	1[251]	CAACGGCGCCAAAGACGCAAAGACACCATTAGGATCCTATTT
4[160]	11[160]	ATCAATAACTCATCGTCAATAACCTGTTATACAAATTCTTAC
16[160]	26[147]	TTTCAACTAATGTGCCTGGAGTGACTCTGGCGAGAAAAACGT
20[48]	20[49]	TGCATTAACGGGCAACAAACAATAACGGATTCATTTCCCAGC
11[35]	4[35]	AAACGGGTAAAAATCGGTGTACAGACGGATCGGTTTGCGAAT
17[238]	15[251]	AGAGAGGTCGTTAGGCGCTTAATGCGCCGCGCACGACGGCCA
6[48]	6[49]	GGTGAATGGTCGCTGAGCTCATTCAGTGATCATTGTGTTCGA
7[28]	8[42]	TAACCGAAAGGCCGGACAGCATCCAGGCGCATAGGCTTGCAG
16[153]	21[153]	GCAAGGAAAGAATTATAATTATAATTTCAGTATTACTAAAAC
10[153]	9[139]	CATATTTGAATATAAGACGACGACAATAAACAATAAGCCAGT
13[119]	3[132]	TGATTCCCCACCCTATAACCCTCGTTTAGCTTTTGTAAGAAC
16[244]	26[231]	AAGTTGGTAAAACGACTTAAGTGTCCTTACCCGCCAACTTGC
7[112]	8[126]	GAAAGACGCGAACCCCTTTTGATGAGGCATTTTCGATTCGAG
27[119]	14[133]	GAGCTTGTGCCATCGCTGAGAAGCATATATTTTAACGACAGT
25[224]	22[224]	GTAATAACATCATCTTTTATAATTAAATGGTGTAGATTCAGC
12[181]	1[195]	GCATGATTATTACGCAGTATGGTATAGCTATAGAAGGCTTAT
2[76]	1[62]	TCAGTTGATACATATAGCAAGCCCAATAGGAACCCTTCAACT
27[35]	14[49]	GTGAACCACTCTGAACCTCGATAAAGACGGAGGATAATCATT
22[125]	24[112]	ATCAACACAATCAACGCTGAGAGCCAGCAGAACGCTTTAGAA
6[76]	12[77]	TAATTTCTTTAAGATGACCAATTAGCCGGAACGAGCGGAGAT
7[70]	5[83]	CTTGCCCATTACCCTAATCTTGACAAGAACCGAACACTGGCT
18[244]	25[244]	AATCTACAAAGGCTCATATGTACCCCCGGAAGTGTAGAGCGG
27[224]	17[237]	CCACCACAGTGCTGCAGTCACGACGTTGGTAACGCATTTTTG
2[97]	13[97]	AGTAAAATACGAGGCACCACCCTCATTTACACGGTGTCTGGA
12[41]	0[28]	AATGCCAAGTGAGATTCCAGACGTTAGTCCTCATACGTCACC
13[224]	3[237]	AACATATTCAGTACATAAACAGTTAATGGTTTTAAATCTTAC
14[174]	23[174]	CTATTTAAGCGAAACGCCAGCCTACATTCATTGGCATAGAAC
10[125]	10[126]	AGGCAAAGAGGTCAATATAATGCGCTGAAAAGGTGGTAATTT
2[118]	12[105]	AGTTTTGCACTATCCAGAACCGCCACCCTTCCATAACATGTT
8[250]	11[244]	AGACTGCGGAACCCCCTCAGAGCCACCGGAGGTTAGCAAAAGACGGAA
3[133]	1[153]	GGGTATTATTTTCAGCGAACGAGTAGATGTACCGCACGATAAAAATCAT
25[56]	18[49]	TCTCAGATGATGGCATTATTTGCACGTGTTACCTGTTTTAATGAGCAT
23[91]	21[111]	AGGTTATCTAAAATTGAATAACAATCGCAAGACAAAGCAAATAAATATC
11[105]	7[111]	TTGCTGATTTTTGCAATTGCTAGACCGGAAGCAAAGTAAGAGGAAGCCC
2[195]	7[202]	AACCTCCAGATTAGAGCCATATTTGTTTAGAGAATACAAAGTCAGAGGG
27[161]	18[168]	GGAAGAACGCTCGCTAGGTAAAGATTCAGGGAGAAATAAAGCATAAGAA
4[237]	9[244]	GAGCGTCATTGACAACCCTCAATAATCAAAATCACTAGCGCGTGAAACC
21[238]	18[245]	GTAACCGTCACGTTGTGAGCGAGTAACAATCCTGAGCACCGCTGTTGGG
21[28]	18[35]	TTTTCACCCGCCTGTGGTTTGCCCCAGCATCGGCAAGTCGGGAAAGCCT
2[216]	11[223]	TGAGTGCCCAGCTATTTGCCAAACACCAGAGCCGCATTACCAGAGGGAG
16[83]	26[63]	TTTTTTGAGTGAATAATTTTCTCGGCTGACGCATTGGGGTCGTATTCCT
27[203]	22[210]	TAGCGGTCACGCTGTCAAACTTACTTCTCGAGTAACTGTAGCTTAACCA
15[252]	21[258]	GTGCCAAGCTGCAAGTGCGGGGCGCAACTTCTGGTACTCCAGGCCAGTT
4[97]	11[104]	AAACAGTTAGACTGTGCAACTAAAGTAAGCGCAGACGGTCAGAGCTTAA
18[69]	23[76]	ATTACCTAGCAAAAGCGAATTATTCGCCTGATTGCAGTAACAAAAGAAA
7[182]	9[174]	ACCCTGAAACATAAAAACAGGATAAGAAAGAACAGTAGGGCTGGTTAAG
21[154]	18[161]	ATCGCCATGAATGGTCTGGCCAACAGAGAGATTCAGACCTAATAAACAC
2[160]	10[154]	TTCATCGTAGGAACGTACCGCATCGGCTATAATATCCCATCCGAATCGC
0[237]	8[231]	GTTTTGCAAAAGAAACAAAAGAAATATTTCACCAGTCACCAATTTTCAT
7[203]	11[209]	TAATTTTAGCAGCCTTTACAGAACGTCACCCTTTTACCAGAAGGAAACC

14[258]	22[252]	AATTCATGCTACAGATAACGTACAGGAGCGCCAGAACCCGTCCGTAATG
25[35]	14[28]	TGTTCCAGGACTCCACACAACCGAGCTCGAATTCGGCTTGTTCCTCCTG
25[182]	14[175]	TCAATCGCAGAACACATAAAGTGTAATACTTTTGCAAAGGGTTTCTAAT
1[252]	7[258]	CGGAACCACAGGAGCTGAATTAAAGCCAGTCAGACACCGCCAAGAGCCA
2[223]	1[216]	AGTGCCTTGCCCGTCAGGCGGATAAGTGCCGTCGATCTAAGAACAACAG
8[230]	7[223]	CGGCATTTCTTTTCGAGCCGCCACCAGAACCACATATTAGCGTTTGCCA
22[41]	25[34]	CCCTTCACAGTGAGATGAATCGGCCAACGCTTTCCAAATCCCTGAGTGT
14[48]	22[42]	TCTCCGAATCACCCAGAACGTGTTTGGATTCCGAAAGGCGAACTGATTG
11[245]	0[238]	ATTATTCTACCAGCGCAGTCTTGTACTGGTAATAACCCCCTGTAGCGGG
14[132]	22[126]	GCGGCCCACGGGGAGTTTGAGACAACTCAATACATTAATAGATTGGCAA
21[217]	18[224]	ATTTTTGTCATTTTCAGCTTTCATCAACATCAGTGTGTCAATATCAGGT
1[63]	7[69]	AATGCAGAGATTTAAACTAACAAGAAAAATGCGATAACTTTAAATAAGG
27[182]	18[189]	GCGCTAGACCCCGCGAGAAAGGCCGGAGATGACCCCTAAATCAGGTGAT
25[119]	14[112]	CAATTCGTAACATTACATCCATCAATAGTGAATTTTTAAGACTGTAAGC
14[90]	22[84]	ATAGGGGACTAAATGAAGGAGATCCTGAGTTAGAAGATTTTCTCAGATG
12[104]	6[98]	TTAAATAGATAGCGTCCAATACCCTCAACATAAATATCAAAAAGATTAG
8[41]	11[34]	GGAGTTATATATTCTTCTTAAACAGCTTTAATTGTAACGAGGTTCCATT
9[161]	6[154]	GACAAAATAATTGATAATTTACGAGCAAGAAGCGCATTAGTTTATCAAC
21[112]	18[119]	AAACCCTGTTGAAAGGAGCACTAACAACTTGAGGAGAGAAAATAGTAGC
2[55]	13[62]	CAGTTTTGTCGTCTATAGAAAGGAACAATGTGTCGGACCCCCAGCGATT
0[258]	8[251]	AGAAGGACGGAATATATGGTTATTAAAGTGGGAATGCAGCACTTAGCGTC
13[63]	8[63]	ATACCAAATCGCCTATGTTACCTTTGAATCAAGAGAAATCAA
15[217]	15[209]	TGATACCAATTGTCAACCTTATGACAATAATCACC
11[140]	2[140]	ATGCGTTTAGCTATAAACCAACAAAATA AAA AAA AAA A
18[160]	27[160]	CGGAATCAGCAAAAACAGGAAAAGGAAG AAA AAA AAA A
20[97]	25[97]	CTAAAGCAAGAAAATGGAAGGTTGTTTG AAA AAA AAA A
3[77]	4[77]	CATTATTACAGGTTTCTACCAGTCAGGA AAA AAA AAA A
4[181]	2[182]	TCCCAATGAAGCCTTAAATCACGACTTG AAA AAA AAA A
9[140]	4[140]	AATAAGAAACAACGAAGAAAAGTCTTTC AAA AAA AAA A
20[76]	25[76]	CGCAGAGGAAGATGTATCAAAAATTCAT AAA AAA AAA A
18[202]	27[202]	AAACAAGGGTTGTATTGCTGGGCAAGTG AAA AAA AAA A
18[223]	27[223]	CATTGCCCGGAGAGGAAGAACCGCGTAA AAA AAA AAA A
11[224]	2[224]	GGAAGGTGGCGACAATCCTGACGGGGTC AAA AAA AAA A
18[48]	25[55]	AAAGTGTAAACCTGTCGTGAAATGGTGGACAAGAG AAA AAA AAA A
16[132]	27[139]	ACCCTAGATAAATCATACAGGTTTAAAAAAGCCGG AAA AAA AAA A
9[91]	4[98]	TAGAGAGTACCTTTGGATGGCGAATGACATGCTTT AAA AAA AAA A
13[210]	2[203]	TACATAAGAAACGCAATAATATTTGCACGAGGCGT AAA AAA AAA A
18[83]	27[76]	ACAATTTCATTTGACAATATAATCATCAAGGTGCC AAA AAA AAA A
11[210]	4[203]	GAGGATTTTATCTTACCGAAGAAAATGAGTTACAA AAA AAA AAA A
20[188]	25[181]	AATTGAGCAGAAGAGGTTTGATTATTTATTGACGC AAA AAA AAA A
13[126]	2[119]	CAATTCTTTTGGGGCGCGATGATCGTCACAAAAGA AAA AAA AAA A
14[195]	25[202]	CAAAATAGGCGCTGTAATATCTCAAATTAACCGTT AAA AAA AAA A
18[188]	27[181]	AAATAAGGCGTTAACTCAGAGATATTACGGAGCGG AAA AAA AAA A
14[111]	25[118]	AACTCGTGAGCCCCTGCGGAACTAGACTTTACAAA AAA AAA AAA A
16[48]	27[55]	CGGGTACATACGAGCCGGAGACTATTAAAAATCAA AAA AAA AAA A
9[175]	4[182]	CCCAATAATAAGAGAGTAAGCACGATTTTTATTTA AAA AAA AAA A
21[140]	25[139]	GATAGCCACACCGCCTGCAACAAATATAAATAGATGTATTAA AAA AAA AAA A
10[55]	2[56]	AGATGAACCGCGACCTGCTCCGATAAATTAAAACGGGAATAC AAA AAA AAA A
13[98]	2[98]	AGTTTCATCAGAGCCATAGTAAGAGCAACCAGAGGGGGTAAT AAA AAA AAA A
4[55]	4[56]	TTAACTAAAGGAATTATCAGCTTGCTAATTACCTTATCTACG AAA AAA AAA A
11[161]	4[161]	CAGTATAGCAAATGGAGAACAAGCAATTCCATGTAGAAACCA AAA AAA AAA A
21[224]	25[223]	TTAAAGGGAAAAGCCCCAAAAACTAGCAAGGCCACTTGATTA AAA AAA AAA A
13[77]	2[77]	ACAAAGTTCAGGGAACGCCAAAAGGAATTGTAGAAAGATTCA AAA AAA AAA A
13[161]	2[161]	AGATACATCACCGTCCCAATAGCAAGCATTGCCGTTTTTATT AAA AAA AAA A
8[223]	4[224]	TTCGGTCCAAGGCCGGAAACGTAGCACCCGCCAGCTTTCCAG AAA AAA AAA A
18[118]	27[97]	ATATGCTGATGCAAATCCAAAATTAATTAGGTTGGACCACCACGGAACC AAA AAA AAA A

8[125]	4[119]	CTTCAAATTCAAATGCAAAGCGGATTGCCAAAAATCAGGTCTTATTCAT AAA AAA AAA A
22[167]	25[160]	ATATTTTTTAAAAAGAGGTGAGGCGGTCATCTTCTCCAGTCAGCTCATG AAA AAA AAA A
25[98]	27[118]	GATTATACAAAGAAGTTATATAACTATATGTAATAATCATTTCGATTTA AAA AAA AAA A

Table S2. Components of buffers.

Buffer	Component
DNA origami folding buffer	TE (1X) +$MgCl_2$ (20 mM) +NaCl (5 mM)
DNA origami wash buffer	TBE (0.5X) +$MgCl_2$ (5 mM)
AuNR-DNA wash buffer	TBE (0.5X) +SDS (0.1%)
Assembly buffer	TBE (1X) + $MgCl_2$ (10 mM) +NaCl (500 mM) +SDS (0.05%)
Gel running buffer	TBE (0.5X) +$MgCl_2$ (11 mM)
Incorporation buffer	TBE (0.5X) + $MgCl_2$ (20 mM) +NaCl (500 mM) +SDS (0.02%)
Reaction buffer	PBS (1X) + $MgCl_2$ (5 mM) +SDS (0.02%)

Table S3. Docking related strands.

Name	Sequence (5'-3')
Staple with docking 1	GTTCGCTCGCTAG TT CGTAACAAAGCTGGCTGGCTGACCTTCAAGAGGAC
Staple with docking 2	TGAGAGTTCGTAAAACAGGAAGCATTAA TT CTCTGCTCACTCT
tail 1	CTAGCGAGCGAAC
tail 2	AGAGTGAGCAGAG

Table S4. Oligonucleotides of interest and competing strands.

Name	DNA/RNA	Free energy of secondary structure (kcal/mol)	Length (nt)	Sequence (5'-3')
Apt1-tail	DNA	-1.46	15+16	CTTGGGTGAGTTCAA ATA AGAGTGAGCAGAG
Apt1R-tail	chimera	NA	15+16	CUUGGGUGAGUUCAA ATA AGAGTGAGCAGAG
CS1-tail	DNA	-1.25	10+16	CTAGCGAGCGAAC ATA TTGAACTCAC
CS1R-tail	DNA	-1.25	9+16	CTAGCGAGCGAAC ATA TTGAACTCA
Apt2-tail	DNA	-3.58	15+16	CTAGCGAGCGAAC ATA GTGAGTTCAA CTTGG
CS2-tail	DNA	-5.15	10+16	TTGAACTCAC ATA AGAGTGAGCAGAG
ST5	DNA	0	11	ACTCACCCAAG
ST4	DNA	0	11	AACTCACCCAA
ST3	DNA	0	11	GAACTCACCCA
ST2	DNA	0	11	TGAACTCACCC
ST1	DNA	0	11	TTGAACTCACC

*The free energies were generated by NUPACK (25°C, default condition)

Table S5. Aptamers from the literature.

Name	Sequence	Length (nt)
ATP RNA Aptamer[1,2]	GGGUUGGGAAGAAACUGCGGCACUUCGGUGCCGGCAACCC	40
Glucose DNA Aptamer[3]	ACGACCGTGTGTGTTGCTCTGTAACAGTGTCCATTGTCGT	40

Table S6. Aptamers and complementary strands used in the paper.

Name	DNA/RNA	Free energy of secondary structure (kcal/mol)*	Length (nt)	Sequence (5'-3')

5'Glucose aptamer	DNA	NA	40+16	CTAGCGAGCGAAC TTT ACG ACC GTG TGT GTT GCT CTG TAA CAG TGT CCA TTG TCG T
3'Glucose aptamer	DNA	NA	40+16	ACG ACC GTG TGT GTT GCT CTG TAA CAG TGT CCA TTG TCG T TTT AGAGTG AGCAGAG
gluCS1	DNA	-2.40	10+16	ACACGGTCGT TTT AGAGTGAGCAGAG
gluCS2	DNA	-5.92	10+16	AGAGCAACAC TTT AGAGTGAGCAGAG
gluCS3	DNA	-1.41	10+16	CTAGCGAGCGAAC TTT ACGACAATGG
gluS	DNA	0	10	ACACACACGG
open strand for glucose aptamer	DNA	0	15	ACACACACGGTCGT
5'ATP aptamer	chimera	NA	40+16	CTAGCGAGCGAAC ATA GGG UUG GGA AGA AAC UGC GGC ACU UCG GUG CCG GCA ACC C
3'ATP aptamer	chimera	NA	40+16	GGG UUG GGA AGA AAC UGC GGC ACU UCG GUG CCG GCA ACC C AAA AGAGTGAGCAGAG
ATPCS1	DNA	-0.08	9+16	TCCCAACCC AAA AGAGTGAGCAGAG
ATPCS2	DNA	-0.67	9+16	GCAGTTTCT AAA AGAGTGAGCAGAG
ATPCS3	DNA	-2.81	9+16	CTAGCGAGCGAAC AAA GGGTTGCCG
ATPS	RNA	0	10	UUUCUUCCCA
open strand for ATP aptamer	DNA	0	15	GTTTCTTCCCAACCC

*The free energy was for the aptamer or complementary strand interacting with the added tail sequence.

2. Methods

DNA origami assembly:

Core staples were mixed with docking staples at 1:1.5 ratio to obtain the staple solutions. For 1-step method, the oligonucleotides of interests (ONI) were added with the 2 times concentration of core staples. Scaffold strand (10 nM) and staples were mixed at 1:10 ratio in DNA origami folding buffer and then assembled using the established protocol by thermal annealing from 80˚C to 20˚C.[4,5] The DNA origami solution was then purified in wash buffer using spin filters (cut off size 100 kDa) for 3 times to remove the free staple strands following the protocol provided by the manufacture.

Gold nanorods (AuNRs) functionalization:

The AuNRs were synthesized using the previously published protocol[5] and functionalized with 5'-thiol-TTTTTT TTTTTT T-3' DNA strands by freeze–thaw method[6]. Bare AuNRs were mixed with thiol-polyT DNA strands at 1:10000 ratio. The mixture was supplemented with 0.05% SDS and frozen for 1h at -20˚C. The AuNR-DNA were washed by centrifugation at 7000 rcf for 30min in wash buffer for 4 times to remove free thiol- polyT DNA strands.

DNA origami-AuNRs assembly:

The DNA origami and AuNR-DNA were mixed at 1:15 ratio and annealed from 40˚C to 20˚C in assembly buffer. The DNA origami-AuNRs with free AuNR-DNA was either directly used for the next step or purified through gel electrophoresis in 0.7% agarose gel for 3h at 80 V.

Incorporation of oligonucleotides of interest (ONI):

The concentration of purified DNA origami-AuNRs was calculated by measuring its absorption value (peak ~650 nm) and dividing by the estimated extinction coefficient of 3.4 $nM^{-1}\cdot cm^{-1}$. The concentration of unpurified DNA origami-AuNRs was assumed to be equal to the input DNA origami concentration as the product output yield was generally very high. The DNA origami-AuNRs were mixed with the ONI in incorporation buffer. The ratio of ONI (comprised of the aptamer and the complementary strand, sequences in SI Tables S4, S6) and the purified DNA origami-AuNRs (~0.3 nM) or non-purified DNA origami-AuNRs (~0.5 nM) was varied between

2:1 and 250:1. The samples were either incubated in a shaker (300 rpm) at room temperature for overnight or annealed from 42˚C to 20˚C with temperature ramp of 0.1 °C min^{-1} between the steps.

temperature (°C)	time (h)
42	2
36	2
30	2
20	hold

Transmission electron microscopes (TEM) characterization:

The samples were deposited on glow-discharged TEM grids (300 mesh formvar/carbon-coated copper) and imaged using FEI Tecnai F12 TEM operating at 120 kV.

Sample preparation for measuring circular dichroism (CD) signal:

To compare different DNA origami-AuNRs samples fabricated with different workflows, 3 completely independent experiments were conducted, during which DNA origami folding, purification, DNA functionalization of AuNRs, and the DNA origami-AuNRs assembly and purification were all performed separately.

For kinetic experiments, 7 µL solutions of the ONI-DNA origami-AuNRs probes were added to the 63 µL reaction buffer containing reference analyte or target and mixed by vortex. The 70 µL samples were immediately pipetted into the cuvette (within 30 seconds) and the CD amplitude at 620 nm was recorded with the Jasco J-1500 CD spectrometer. The CD amplitude of the aliquot ONI-DNA origami-AuNRs sample that underwent the same operation in the reaction buffer without target or reference analyte was used as the control points before target binding.

For thermodynamic experiments, the samples of ONI-DNA origami-AuNRs were incubated in 70 µL reaction buffer containing different concentration of reference analyte or target at room temperature with shaking at 100 rpm. The CD spectra were measured after overnight incubation.

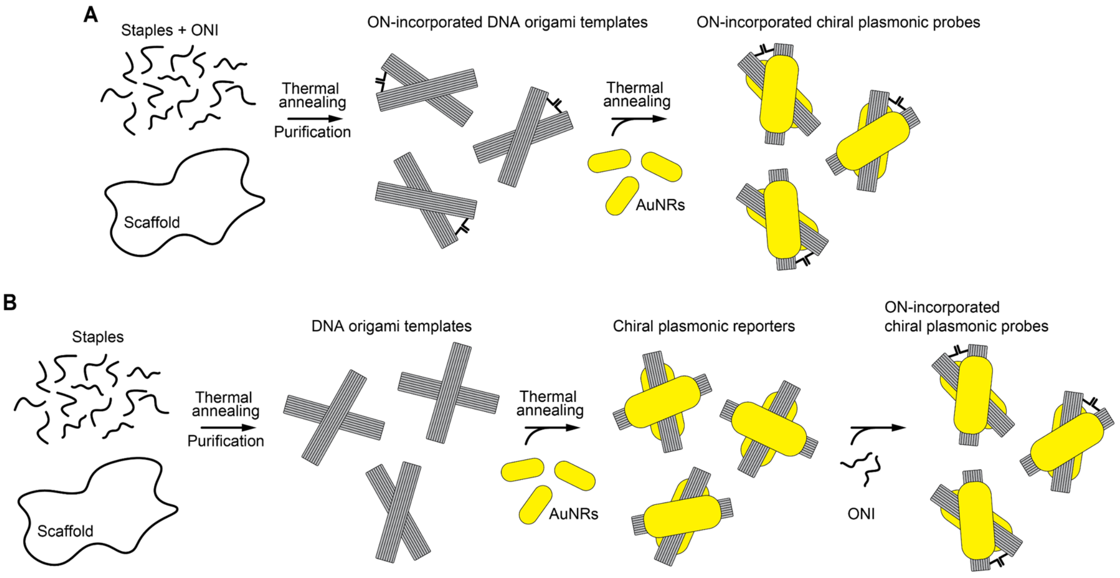

Figure S1. Incorporation of ONI into the DNA origami-AuNRs reporters using different methods. **(A)** 1-step method. **(B)** 2-step method.

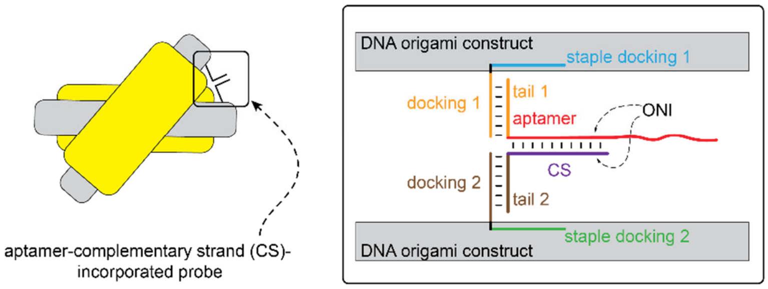

Figure S2. The docking sequences on DNA origami hybridize with the tail sequences on ONI.

3. Design benefits

As the targets of aptamers involve a broad range of molecules and the K_D values vary from picomolar to millimolar, partitioning bound and unbound relying on the physical and chemical characteristic of particular aptamer-target pair demands significant efforts. In addition, methods for obtaining insights on functional domains for developing applications (e.g., biosensors), such as point mutation of the sequence, can be expensive. The competitive hybridization-based strategy can combine the domain investigation and K_D measurement and, in principle, be applicable to most of the aptamers including DNA, RNA, and modified nucleotides, as long as the aptamers can form duplex with DNA/RNA or with themselves (the complementary strand and reference analyte are adjusted accordingly). The base-paring of common artificial nucleic acids with synthetic backbones (e.g., LNA, PNA, SNA and ZNA) follows similar predictable rules of DNA and RNA, although changes in the thermodynamic and kinetic parameters must be considered. The method is also valid for the aptamers composed of modified bases which are designed to maintain canonical base-pairing (e.g., SOMAmers). The competitive hybridization-based approach, however, may not apply to some aptamers with extensive intramolecular hybridization, as the complementary strand may act as a partial aptamer. To solve the limitation, a split aptamer strategy may be used instead.[7]

The affinity of an aptamer-target can be controversial when using different measurement techniques, especially for small molecules [8–10], while the binding strengths of nucleic acids with given sequences are often widely agreed. Although, the hybridization energies may require calibration for different systems, the K_D of two oligonucleotides usually can be measured robustly. By introducing the DNA/RNA reference analyte and relative K_D, we provide a solution to address the discrepancy in the reported affinities of aptamers by converting the inconsistent values into a commonly agreed value.

Compared to Förster resonance energy transfer (FRET), where the signal is correlated with the distance of the donor and acceptor, the CD signal is determined by the chiral configuration of the probe.[4,11] Therefore, the aptamer and the complementary strand can be confined in a small volume and the background noise signals

generated by the proximity of two stands can be avoided in the CD probes. Thus, the high local concentration of the aptamer and complementary strand, which is tolerated by CD probes, drives the equilibrium to the hybridized state, so even a weak hybridization with large dissociation rate constant can produce a strong signal for accurate quantification. Also, in the FRET-based systems, the hybridization is preferred at the ends of the two strands, where the donor and the acceptor are typically placed. The CD-based probes allow the hybridization in the middle of the sequence, enabling the investigation of the kinetic behaviors of different blocking regions.

The insertion of aptamers and complementary strands after the fabrication of the DNA origami-AuNRs reporters (2-step fabrication method) reduces the possibilities of deactivating unstable nucleic acid strands (e.g., some RNA). Also, compared to the typical 1-step method, where the probe diversities are already generated at the initial step, incorporating different aptamers at the final stage for distinct functionalities enables large scale production of unified DNA origami-AuNRs reporters.

4. Results

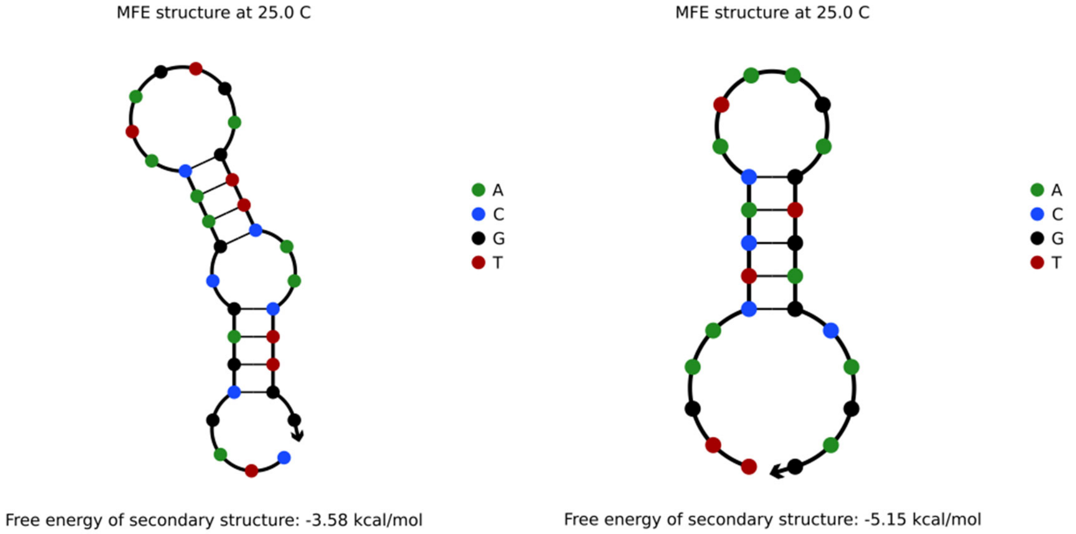

Figure S3. The secondary structure of the Apt2 with the tail added to 5' end (left) and the CS2 with the tail added to the 3' end (right), generated by NUPACK.[12]

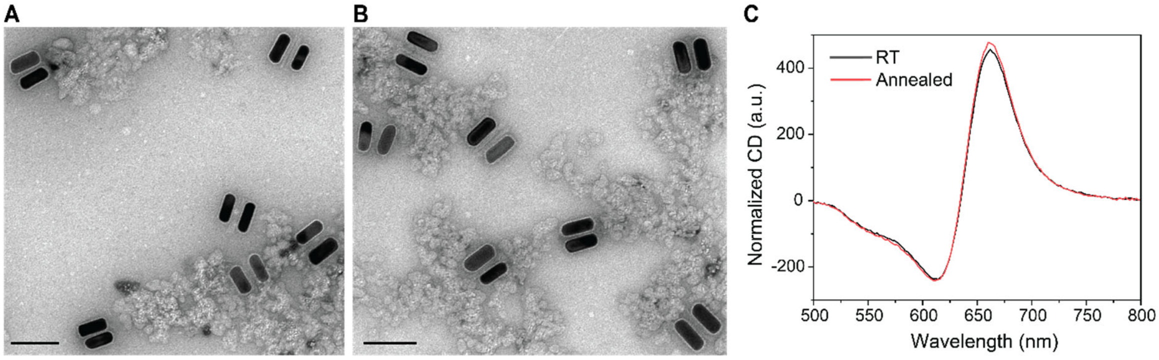

Figure S4. **(A, B)** The TEM image of the probes of DNA origami-AuNRs after annealing **(A)** and room temperature (RT) incubation **(B)**. Scale bar represents 100 nm. **(C)** The CD spectra of the reporter after annealing and room temperature incubation without ONI.

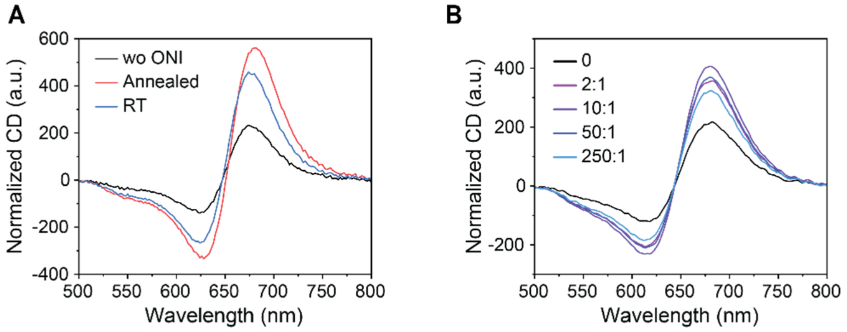

Figure S5. **(A, B)** The CD spectra of purified probe of DNA origami-AuNRs incorporated by the Apt1R and CSR1 (9nt). The effects of the temperature **(A)** (the concentration ratio of ONI and reporter was fixed at 50:1) and the concentration ratio of the ONI and the DNA origami **(B)** (with annealing treatment).

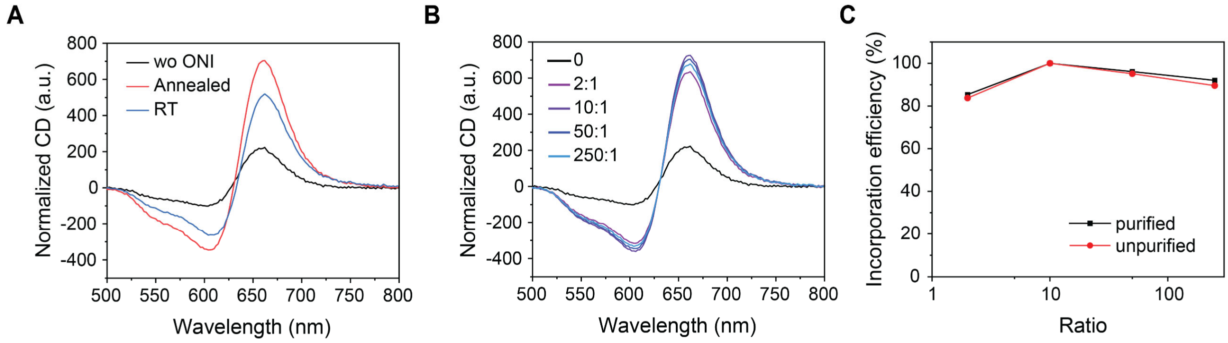

Figure S6. **(A, B)** The CD spectra of unpurified probes of DNA origami-AuNRs incorporated by the ONI-1. The effects of the temperature **(A)** (the concentration ratio of ONI and reporter was fixed at 50:1) and the concentration ratio of the ONI and the DNA origami **(B)** (with annealing treatment). **(C)** The comparison of ratio effects between the purified and unpurified samples.

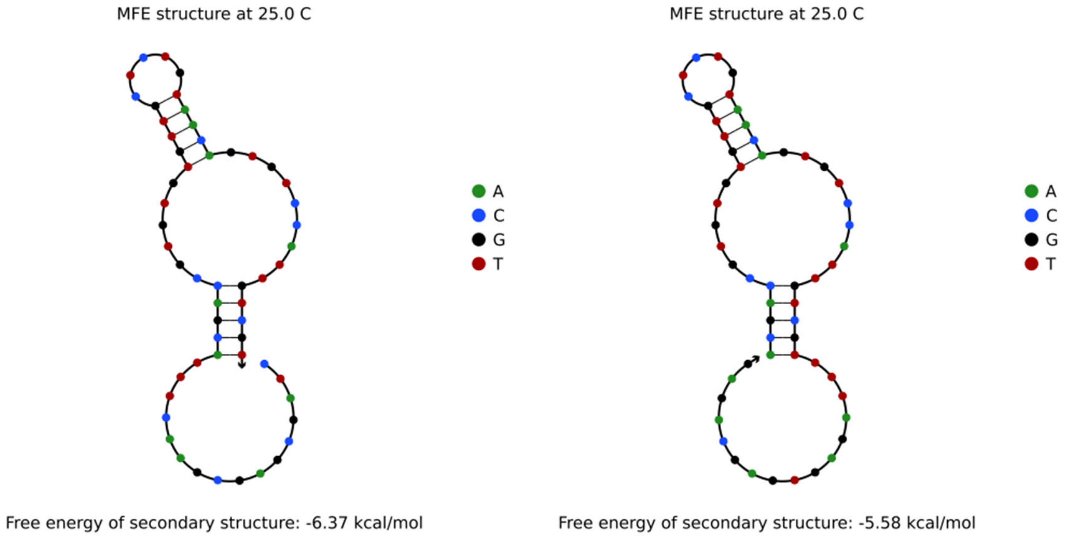

Figure S7. The secondary structure of the glucose DNA aptamer with the tail added to the 5' end (left) and the 3' end (right), generated by NUPACK.[12]

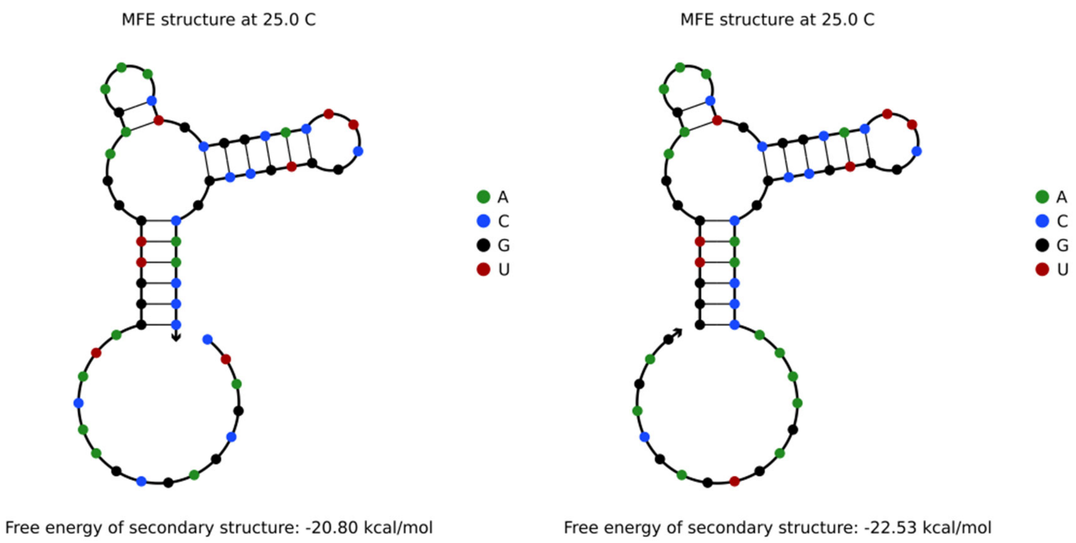

Figure S8. The secondary structure of the ATP RNA aptamer with the tail added to the 5' end (left) and 3' end (right), generated by NUPACK (DNA tails were altered to RNA tails as chimera is unavailable).[12]

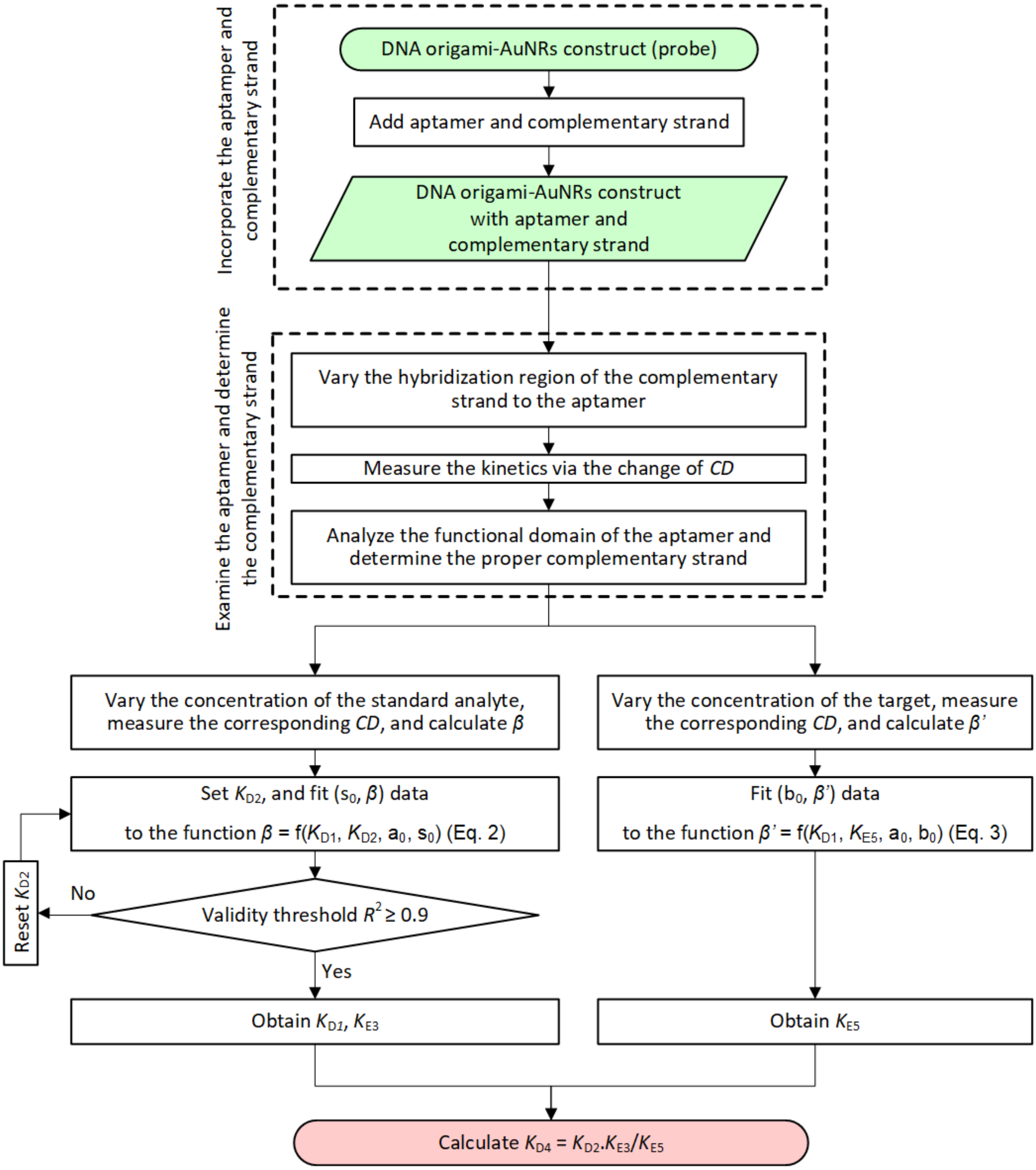

Figure S9. Workflow for measuring aptamer affinities.

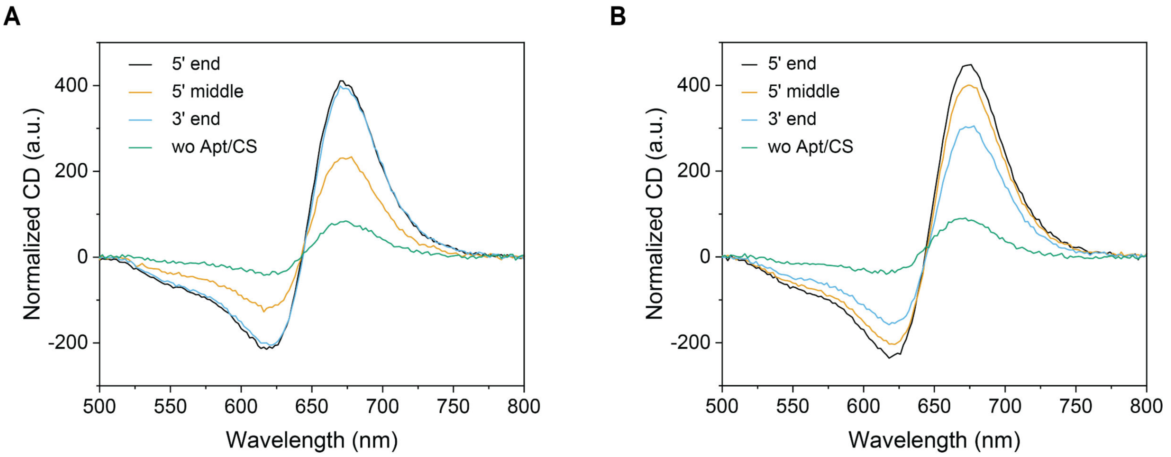

Figure S10.The CD spectra of the probes incorporated with different pairs of aptamer-complementary strand. **(A)** glucose aptamer-gluCS(1-3) and **(B)** ATP aptamer-ATPCS(1-3).

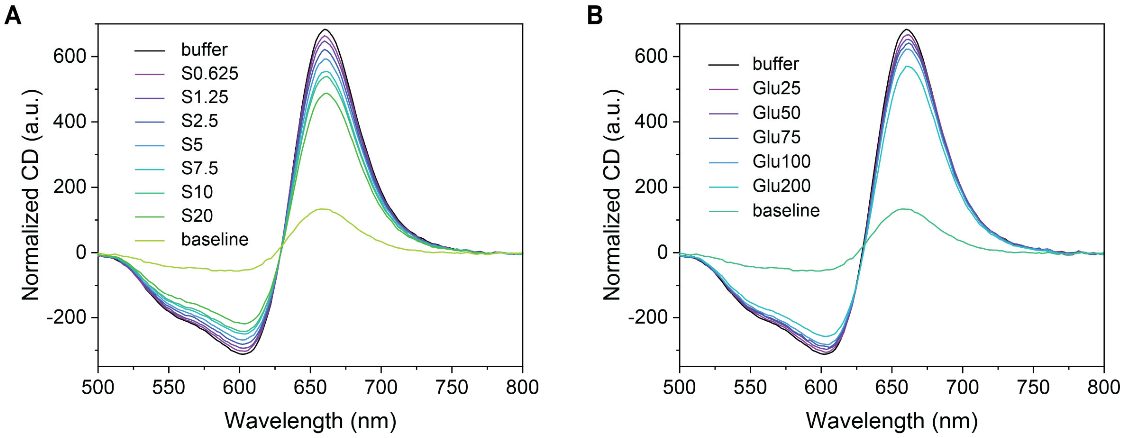

Figure S11. The CD spectra of the glucose aptamer-complementary strand (gluCS1) incubated with different concentration of the reference analyte **(A)** and glucose **(B)**.

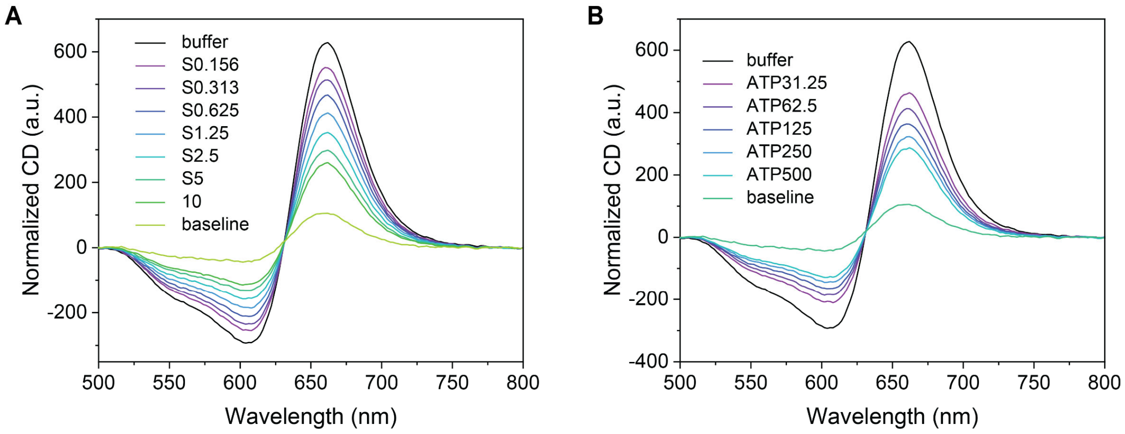

Figure S12. The CD spectra of the ATP aptamer-complementary strand (ATPCS1) incubated with different concentration of the reference analyte **(A)** and ATP **(B)**.

5. Theoretical model

Concentrations at equilibrium

In the samples without reference analytes or targets, the reaction 1 is at equilibrium; in the samples with reference analytes, reaction 1, 2, and 3 are at equilibrium; in the samples with targets, reaction 1, 4, 5 are at equilibrium.

$$\mathrm{A} + \mathrm{C} \overset{K_{\mathrm{D1}}}{\longleftrightarrow} \mathrm{AC} \qquad \text{(reaction 1)}$$

$$\mathrm{A} + \mathrm{S} \overset{K_{\mathrm{D2}}}{\longleftrightarrow} \mathrm{AS} \qquad \text{(reaction 2)}$$

$$\mathrm{AC} + \mathrm{S} \overset{K_{\mathrm{E3}}}{\longleftrightarrow} \mathrm{AS} + \mathrm{C} \qquad \text{(reaction 3)}$$

$$\mathrm{A} + \mathrm{T} \overset{K_{\mathrm{D4}}}{\longleftrightarrow} \mathrm{AT} \qquad \text{(reaction 4)}$$

$$\mathrm{AC} + \mathrm{T} \overset{K_{\mathrm{E5}}}{\longleftrightarrow} \mathrm{AT} + \mathrm{C} \qquad \text{(reaction 5)}$$

where, K_{D1}, K_{D2}, and K_{D4} are the dissociation constants of the aptamer-complementary strand (AC), the aptamer-reference analyte (AS), and the aptamer-target (AT), respectively; K_{E3} and K_{E5} are the equilibrium constants of the displacement reactions 3 and 5, where $K_{\mathrm{E3}} = \frac{K_{\mathrm{D1}}}{K_{\mathrm{D2}}}$ and $K_{\mathrm{E5}} = \frac{K_{\mathrm{D1}}}{K_{\mathrm{D4}}}$, respectively; a_0 is the input local concentration of the aptamer/complementary strand; b_0 and s_0 are the input bulk concentration of the targets and the reference analytes, respectively. The concentrations of the targets and reference analytes remain constant as the input bulk concentrations (hundreds of nanomolar to millimolar in our experiments) are much higher than the bulk concentration of the probes (tens of picomolar).

Table S7. The concentration of each species at equilibrium.

	[A]	[T]/ [S]	[C]	[AT]/ [AS]	[AC]
**Equilibrium state without target/reference analyte**	$a_0 - x$	0	$a_0 - x$	0	x
**Equilibrium state with reference analyte**	$a_0 - y - z$	s_0	$a_0 - y$	z	y
**Equilibrium state with target**	$a_0 - y' - z'$	b_0	$a_0 - y'$	z'	y'

$$K_{\mathrm{D1}} = \frac{[\mathrm{A}]\cdot[\mathrm{C}]}{[\mathrm{AC}]} \qquad \text{(Eq. S1)}$$

$$K_{\mathrm{D2}} = \frac{[\mathrm{A}]\cdot[\mathrm{S}]}{[\mathrm{AS}]} \qquad \text{(Eq. S2)}$$

$$K_{\mathrm{E3}} = \frac{[\mathrm{AS}]\cdot[\mathrm{C}]}{[\mathrm{AC}]\cdot[\mathrm{S}]} \qquad \text{(Eq. S3)}$$

$$K_{\mathrm{D4}} = \frac{[\mathrm{A}]\cdot[\mathrm{T}]}{[\mathrm{AT}]} \qquad \text{(Eq. S4)}$$

$$K_{\mathrm{E5}} = \frac{[\mathrm{AT}]\cdot[\mathrm{C}]}{[\mathrm{AC}]\cdot[\mathrm{T}]} \qquad \text{(Eq. S5)}$$

Substitute the concentration into the equations S1 and S2. Therefore,

$$K_{D1} = \frac{(a_0-x)^2}{x} \qquad \text{(Eq. S6)}$$

$$K_{D1} = \frac{(a_0-y-z)\cdot(a_0-y)}{y} = \frac{(a_0-y'-z')\cdot(a_0-y')}{y'} \qquad \text{(Eq. S7)}$$

$$K_{D2} = \frac{s_0\,(a_0-y-z)}{z} \qquad \text{(Eq. S8)}$$

$$K_{E3} = \frac{z\,(a_0 - y)}{s_0 \cdot y} \quad \text{(Eq. S9)}$$

$$K_{D4} = \frac{b_0\,(a_0 - y\prime - z\prime)}{z\prime} \quad \text{(Eq. S10)}$$

$$K_{E5} = \frac{z\prime\,(a_0 - y\prime)}{b_0 \cdot y\prime} \quad \text{(Eq. S11)}$$

Solve x, y, y', z, and z':

$$x = a_0 + \frac{K_{D1}}{2} - \frac{\sqrt{4a_0K_{D1} + K_{D1}^2}}{2} \quad \text{(Eq. S12)}$$

$$y = \frac{K_{D1} + s_0\frac{K_{D1}}{K_{D2}} + 2a_0 - \sqrt{(K_{D1} + s_0 \cdot \frac{K_{D1}}{K_{D2}})^2 + 4a_0\left(K_{D1} + s_0 \cdot \frac{K_{D1}}{K_{D2}}\right)}}{2} \quad \text{(Eq. S13)}$$

$$y' = \frac{K_{D1} + b_0 \cdot K_{E5} + 2a_0 - \sqrt{(K_{D1} + b_0 \cdot K_{E5})^2 + 4a_0(K_{D1} + b_0 \cdot K_{E5})}}{2} \quad \text{(Eq. S14)}$$

$$z = \frac{K_{D1}}{K_{D2}} \cdot s_0 \cdot \frac{y}{(a_0 - y)} \quad \text{(Eq. S15)}$$

$$z' = \frac{K_{D1}}{K_{D4}} \cdot b_0 \cdot \frac{y\prime}{(a_0 - y\prime)} \quad \text{(Eq. S16)}$$

We define β as the ratio of the AC concentrations in the presence and absence of the reference analyte; β' as the ratio of the AC concentrations in the presence and absence of the target:

$$\beta = \frac{y}{x} = \frac{s_0\frac{K_{D1}}{d} + K_{D1} + 2a_0 - \sqrt{\left(1 + \frac{s_0}{d}\right)^2 K_{D1}{}^2 + 4a_0\left(1 + \frac{s_0}{d}\right)K_{D1}}}{2a_0 + K_{D1} - \sqrt{4a_0K_{D1} + K_{D1}^2}} \quad \text{(Eq. S17)}$$

$$\beta' = \frac{y\prime}{x\prime} = \frac{b_0K_{E5} + K_{D1} + 2a_0 - \sqrt{(K_{D1} + b_0K_{E5})^2 + 4a_0(K_{D1} + b_0K_{E5})}}{2a_0 + K_{D1} - \sqrt{4a_0K_{D1} + K_{D1}^2}} \quad \text{(Eq. S18)}$$

$$K_{E3} = \frac{K_{D1}}{d} \quad \text{(Eq. S19)}$$

$$K_{D4} = K_{D2} \cdot \frac{K_{E3}}{K_{E5}} \quad \text{(Eq. S20)}$$

where, $d = K_{\mathrm{D2}}$, which is an unknown constant that requires manually adjust and treated as a free parameter. Of note, the obtained $\frac{K_{\mathrm{E3}}}{K_{\mathrm{E5}}}$ values remained similar for a wide range of d. Reasons and detailed examples are given below.

Circular dichroism signals at equilibrium

The measured circular dichroism signal (CD) at the minimum deep wavelength (~620nm), the measured absorption (Abs) at the maximum peak wavelength (~650 nm), and the normalized CD signal (N) can be expressed in terms of concentrations and molar optical coefficients as:

$$CD = c_{closed} \cdot \varepsilon_{CD}^{closed} \cdot l + c_{open} \cdot \varepsilon_{CD}^{open} \cdot l \quad \text{(Eq. S21)}$$

$$Abs = \left(c_{closed} + c_{open}\right) \cdot \varepsilon_{Abs} \cdot l \quad \text{(Eq. S22)}$$

$$N = \frac{CD}{Abs} = \frac{c_{closed} \cdot \varepsilon_{CD}^{closed} + c_{open} \cdot \varepsilon_{CD}^{open}}{(c_{closed} + c_{open}) \cdot \varepsilon_{Abs}} \quad \text{(Eq. S23)}$$

where c_{closed} and c_{open} are the concentrations of the probe of ONI-DNA origami-AuNRs constructs in the chiral (closed) and relaxed (open) configurations, respectively; $\varepsilon_{CD}^{closed}$ and ε_{CD}^{open} are the molar CD signal of the probe in the closed and open configuration, respectively; l is optical path; ε_{Abs} is the molar extinction for the probe, which is irrelative to the configurational state.

The CD, Abs, and N can also be written in terms of the fraction of probes in the closed configuration (f). Thus,

$$CD = c \cdot f \varepsilon_{CD}^{closed} \cdot l + c \cdot (1-f) \cdot \varepsilon_{CD}^{open} \cdot l \quad \text{(Eq. S24)}$$

$$Abs = c \cdot \varepsilon_{Abs} \cdot l \quad \text{(Eq. S25)}$$

$$N = \frac{CD}{Abs} = \frac{f \cdot \varepsilon_{CD}^{closed} + (1-f) \cdot \varepsilon_{CD}^{open}}{\varepsilon_{Abs}} = \frac{f \cdot (\varepsilon_{CD}^{closed} - \varepsilon_{CD}^{open}) + \varepsilon_{CD}^{open}}{\varepsilon_{Abs}} \quad \text{(Eq. S26)}$$

where c is total concentration of the probes in the sample, i.e., $c = c_{closed} + c_{open}$.

when $f = 0$,

$$N_{open} = \frac{CD_{open}}{Abs} = \frac{\varepsilon_{CD}^{open}}{\varepsilon_{Abs}} \quad \text{(Eq. S27)}$$

We term the CD_{open} as baseline CD.

We define the relative CD as CD subtract the baseline and the relative normalized CD signal N^*as:

$$N^* = N - N_{open} = \frac{f \cdot (\varepsilon_{CD}^{closed} - \varepsilon_{CD}^{open}) + \varepsilon_{CD}^{open}}{\varepsilon_{Abs}} - \frac{\varepsilon_{CD}^{open}}{\varepsilon_{Abs}} = f \frac{(\varepsilon_{CD}^{closed} - \varepsilon_{CD}^{open})}{\varepsilon_{Abs}} \quad \text{(Eq. S28)}$$

Therefore, in the absence and presence of the reference analyte/target:

$$N^*_{b_0/s_0=0} = N_{b_0/s_0=0} - N_{open} = \frac{f_{b_0/s_0=0}(\varepsilon_{CD}^{closed} - \varepsilon_{CD}^{open})}{\varepsilon_{Abs}} \quad \text{(Eq. S29)}$$

$$N^*_{s_0} = N_{s_0} - N_{open} = \frac{f_{s_0}(\varepsilon_{CD}^{closed} - \varepsilon_{CD}^{open})}{\varepsilon_{Abs}} \quad \text{(Eq. S30)}$$

$$N^*_{b_0} = N_{b_0} - N_{open} = \frac{f_{b_0}(\varepsilon_{CD}^{closed} - \varepsilon_{CD}^{open})}{\varepsilon_{Abs}} \quad \text{(Eq. S31)}$$

As the closed state of the probe corresponds to the hybridized state of aptamer-complementary stand, the fraction of closed probes equals to the fraction of AC,

$$f = \frac{c_{closed}}{c} = \frac{[AC]}{a_0} \quad \text{(Eq. S32)}$$

Substitute the concentration of AC in the absence and presence of the reference analyte/target:

$$f_{b_0/s_0=0} = \frac{x}{a_0} \quad \text{(Eq. S33)}$$

$$f_{s_0} = \frac{y}{a_0} \quad \text{(Eq. S34)}$$

$$f_{b_0} = \frac{y'}{a_0} \quad \text{(Eq. S35)}$$

Therefore,

$$\frac{N^*_{s_0}}{N^*_{b_0/s_0=0}} = \frac{N_{s_0} - N_{open}}{N_{b_0/s_0=0} - N_{open}} = \left(\frac{f_{s_0}(\varepsilon_{CD}^{closed} - \varepsilon_{CD}^{open})}{\varepsilon_{Abs}} \middle/ \frac{f_{b_0/s_0=0}(\varepsilon_{CD}^{closed} - \varepsilon_{CD}^{open})}{\varepsilon_{Abs}} \right) = \frac{f_{s_0}}{f_{b_0/s_0=0}} = \frac{y}{x} = \beta \quad \text{(Eq. S36)}$$

$$\frac{N^*_{b_0}}{N^*_{b_0/s_0=0}} = \frac{N_{b_0} - N_{open}}{N_{b_0/s_0=0} - N_{open}} = \left(\frac{f_{b_0}(\varepsilon_{CD}^{closed} - \varepsilon_{CD}^{open})}{\varepsilon_{Abs}} \middle/ \frac{f_{b_0/s_0=0}(\varepsilon_{CD}^{closed} - \varepsilon_{CD}^{open})}{\varepsilon_{Abs}} \right) = \frac{f_{b_0}}{f_{b_0/s_0=0}} = \frac{y'}{x} = \beta' \quad \text{(Eq. S37)}$$

Calculation of the input local concentration of aptamer/complementary strand (a_0)

The total local concentration of A and C (a_0) was determined by the geometry of the DNA origami with a defined volume. Here, we made a simplified model to roughly estimate the volume. Based on the persistence length of double stranded DNA (50 nm) and single stranded DNA (2 nm),[13,14] we consider the single stranded A and C are particles on the tubes of the double stranded docking strands (13 bp), anchored on two rigid bundles that can move freely around the fixed pivot point (**Figure S13**).We use the cylinder model for the double strands

with the parameters: length as 0.34 nm/bp and diameter (d) as 2.3 nm to calculate the size of the components.[15] The distance (L) between the pivot point and the docking sites were approximately 26 nm. The distance (h) between the particle and the docking site is approximately 4.4 nm. The two bundles are linked with a single stranded spacer (8 nt) and we estimated the distance (p) between the two bundles as 2 nm (**Figure S13**).

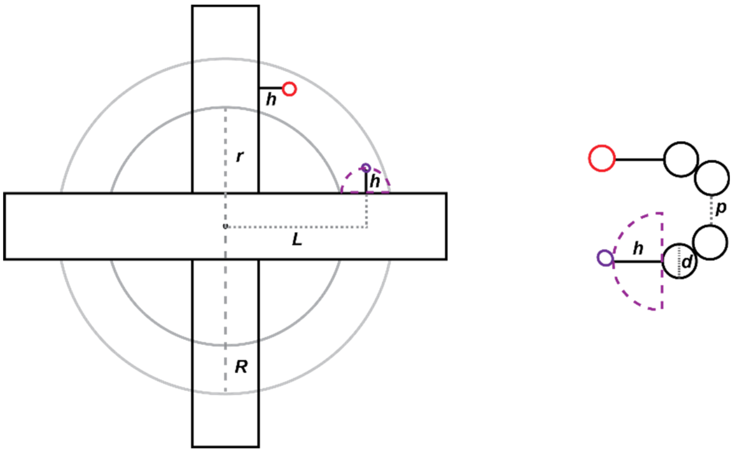

Figure S13. Schematic figure for DNA origami geometry. The top view (left) and the side view (right). To illustrate the length and distance clearly, the size in the side view is scaled two times compared to the size of the top view.

$$R = L + h = 26 \text{ nm} + 4.4 \text{ nm} = 30.4 \text{ nm}$$
$$r = L - h = 26 \text{ nm} - 4.4 \text{ nm} = 21.6 \text{ nm}$$
$$a_0 = \frac{n}{V} = \frac{\frac{1}{Na}}{(\pi R^2 - \pi r^2) \cdot (2h + 2d + p)} = \frac{\frac{1}{6.02 \cdot 10^{23}} \text{ M}}{\pi(30.4^2 - 21.6^2) \times (2 \times 4.4 + 2 \times 2.3 + 2) \text{ nm}^3} \approx 70 \text{ µM}$$

6. Data analysis

Calculation of the dissociation constant of aptamer and analyte

workflow in general:

1. Measure the baseline *CD* and *Abs* to obtain N_{open} using equation S27.
2. Measure the *CD* and *Abs* of the sample in buffer to obtain $N^*_{b_0/s_0=0}$ using equation S29.
3. Vary the concentration of the input reference analytes and measure *CD* and *Abs*. Obtain K_{D1} by fitting β with s_0 using equation S17 by assigning a value to d so the fitting is valid. The coefficient of determination R^2 indicates the goodness of the fitting.
4. Calculate K_{E3} with the obtained K_{D1} and d using the equation S19.
5. Vary the concentration of the input target and obtain K_{E5} by fitting β' with b_0 using equation S18 with the obtained K_{D1}.
6. Calculate K_{D4} as a ratio of K_{D2} with the obtained values of K_{D1}, K_{E3}, and K_{E5} to gain the relative dissociation constant compared to the reference analyte (equation S20).

workflow for DNA glucose aptamer:

1. The probe of DNA origami-AuNRs were incorporated with the glucose aptamer and its complementary strand (10 nt). The sample was incubated with the 15nt open strand (**Table S6**) for overnight to fully

displace the hybridization and to shift the probes into the open configuration. Then, *CD* and *Abs* were measured to calculate baseline $N_{open} = \frac{CD_{open}}{Abs}$.

2. The *CD* and *Abs* of the sample incubated in the buffer were measured to obtain $N^*_{b_0/s_0=0} = N_{b_0/s_0=0} - N_{open}$
3. The concentration of the input reference analyte gluS (10 nt) was varied (0.625 µM, 1.25 µM, 2.5 µM, 5 µM, 7.5 µM, 10 µM, 20 µM) and the corresponding *CD* and *Abs* were measured after overnight incubation.
4. The calculated $\beta = \frac{N^*_{s_0}}{N^*_{b_0/s_0=0}}$ was fitted with s_0 using equation $\beta = \frac{K_{D1}+s_0\frac{K_{D1}}{d}+2a_0-\sqrt{(K_{D1}+s_0\cdot\frac{K_{D1}}{d})^2+4a_0\left(K_{D1}+s_0\cdot\frac{K_{D1}}{d}\right)}}{2a_0+K_{D1}-\sqrt{4a_0K_{D1}+K_{D1}^2}}$, a_0=70 µM, d=1 µM. The dissociation constant K_{D1} was obtained as 1.29 ± 0.0585 µM with R_1^2 =0.992.
5. The equilibrium constant K_{E3} was calculated as 1.29 using $\frac{K_{\mathrm{D1}}}{d}$.
6. The concentration of the input glucose was varied (25 mM, 50 mM, 75 mM, 100 mM, 200 mM) and the corresponding *CD* and *Abs* were measured after overnight incubation.
7. The calculated $\beta' = \frac{N^*_{b_0}}{N^*_{b_0/s_0=0}}$ was fitted with b_0 using equation $\beta' = \frac{K_{D1}+b_0\cdot K_{E5}+2a_0-\sqrt{(K_{D1}+b_0\cdot K_{E5})^2+4a_0(K_{D1}+b_0\cdot K_{E5})}}{2a_0+K_{D1}-\sqrt{4a_0K_{D1}+K_{D1}^2}}$, a_0=70 µM, K_{D1}=1.29 µM.
8. The equilibrium constant K_{E5} was obtained as (3.51 ± 0.295) x 10^{-5} with R_2^2 =0.955.
9. The relative value of $\frac{K_{\mathrm{E3}}}{K_{\mathrm{E5}}}$ was generated as 3.68 x 10^4. The dissociation constant K_{D4} for glucose, thus, was approximately 37000 times of the dissociation constant K_{D2} of aptamer-reference analyte.
10. Here, we manually adjust d to different values to demonstrate the tolerance of the estimation. As shown in the **Table S9**, the relative value $\frac{K_{\mathrm{E3}}}{K_{\mathrm{E5}}}$ remained similar (2.88 x 10^4 – 5.34 x 10^4) when d was varied from 0.01 to 10. We estimate the K_{D2} value between 0.1-1 with the previous knowledge[16] and the variation of relative value was in an acceptable range. The fitting may fail when the d value deviates from the real K_{D2} too much.

Table S8. The fitting values for glucose aptamer.

s₀ (µM)	0,625	1,25	2,5	5	7,5	10	20
β	0,960	0,930	0,879	0,826	0,759	0,728	0,638
b₀ (mM)	25	50	75	100	200		
β'	0,973	0,942	0,917	0,886	0,793		

Table S9. The tolerance of *d*.

d	K_{D1}	R_1^2	K_{E3}	K_{E5}	R_2^2	$\frac{K_{\mathrm{E3}}}{K_{\mathrm{E5}}}$
1	1.29	0.992	1.29	3.51 x 10^{-5}	0.955	**3.68 x 10^4**
0.1	0.0805	0.988	0.805	1.71 x 10^{-5}	0.872	**4.71 x 10^4**
10	48.1	0.931	4.81	1.67 x 10^{-4}	0.995	**2.88 x 10^4**
0.01	0.00683	0.971	0.683	1.28 x 10^{-5}	0.814	**5.34 x 10^4**
100		-0.616 (fitting failed)				
0.001	6.47E-04	0.964	0.647		0.789 (fitting failed)	

workflow for RNA ATP aptamer:

1. The probe of DNA origami-AuNRs were incorporated with the ATP aptamer and its complementary strand (9 nt). The sample was incubated with the 15nt open strand (**Table S6**) for overnight to fully displace the hybridization and to shift the probes into the open configuration. Then, *CD* and *Abs* were measured to calculate baseline $N_{open} = \frac{CD_{open}}{Abs}$.
2. The *CD* and *Abs* of the sample incubated in the buffer were measured to obtain $N^*_{b_0/s_0=0} = N_{b_0/s_0=0} - N_{open}$
3. The concentration of the input reference analyte ATPS (10 nt) was varied (0.156 µM, 0,313 µM, 0.625 µM, 1.25 µM, 2.5 µM, 5 µM, 10 µM) and the corresponding *CD* and *Abs* were measured after overnight incubation.
4. The calculated $\beta = \frac{N^*_{s_0}}{N^*_{b_0/s_0=0}}$ was fitted with s_0 using equation $\beta = \frac{K_{D1}+s_0\frac{K_{D1}}{d}+2a_0-\sqrt{(K_{D1}+s_0\cdot\frac{K_{D1}}{d})^2+4a_0\left(K_{D1}+s_0\cdot\frac{K_{D1}}{d}\right)}}{2a_0+K_{D1}-\sqrt{4a_0K_{D1}+K_{D1}^2}}$, a_0=70 µM, d=0.01 µM. The dissociation constant K_{D1} was obtained as 0.353 ± 0.00982 µM with R_1^2 =0.998.
5. The equilibrium constant K_{E3} was calculated as 35.3 using $\frac{K_{\mathrm{D1}}}{d}$.
6. The concentration of the input ATP was varied (31.3 µM, 62.5 µM, 125 µM, 250 µM, 500 µM) and the corresponding *CD* and *Abs* were measured after overnight incubation.
7. The calculated $\beta' = \frac{N^*_{b_0}}{N^*_{b_0/s_0=0}}$ was fitted with b_0 using equation $\beta' = \frac{K_{D1}+b_0\cdot K_{E5}+2a_0-\sqrt{(K_{D1}+b_0\cdot K_{E5})^2+4a_0(K_{D1}+b_0\cdot K_{E5})}}{2a_0+K_{D1}-\sqrt{4a_0K_{D1}+K_{D1}^2}}$, a_0=70 µM, K_{D1}=0.353 µM.
8. The equilibrium constant K_{E5} was obtained as 0.594 ± 0.0571 with R_2^2 =0.948.
9. The relative value of $\frac{K_{\mathrm{E3}}}{K_{\mathrm{E5}}}$ was generated as 59.5, the dissociation constant K_{D4} for ATP, thus was approximately 60 times of the dissociation constant K_{D2} of the aptamer-reference analyte.
10. Again, we manually adjusted d to different values to demonstrate the tolerance of the estimation. As shown in the **Table S11**, the relative value $\frac{K_{\mathrm{E3}}}{K_{\mathrm{E5}}}$ remained similar (57.8 – 62.6) when d was varied from 0.00001 to 1.

Table S10. The fitting values for ATP aptamer.

s₀ (µM)	0,15625	0,3125	0,625	1,25	2,5	5	10
β	0,821	0,730	0,619	0,495	0,361	0,238	0,150
b₀ (µM)	31,25	62,5	125	250	500		
β'	0,609	0,496	0,399	0,302	0,226		

Table S11. The tolerance of d.

d	K_{D1}	R_1^2	K_{E3}	K_{E5}	R_2^2	$\frac{K_{\mathrm{E3}}}{K_{\mathrm{E5}}}$
0.01	0.353	0.998	35.3	0.594	0.948	**59.5**
0.1	5.56	0.995	55.6	0.912	0.875	**61.0**
0.001	0.0306	0.993	30.6	0.524	0.965	**58.4**
1	757	0.962	757	12.1	0.718	**62.6**
0.0001	0.00292	0.99	29.2	0.504	0.970	**57.9**
0.00001	2.88E-04	0.989	28.8	0.498	0.971	**57.8**

7. MATLAB code

```
% a0 is initial local concentration of AC and b0 is initial bulk concentration of B
% k1, k_1, k2, k_2, k3, and k_3 are rate constants
% KD1, KD2, and KD3 are dissociation constants.
t0 = 0;
tmax =120;
N=100000;
t=linspace(t0,tmax,N);
dt = (tmax-t0)/N;
a0 = 70;
b0 = 1.1;
% b0 decided by input
ConcAB0 = 0;
ConcAB=ConcAB0;
ConcAC0 = 55;
% ConcAC0 decided by hybridization energy.
ConcAC=ConcAC0;
CD=ConcAC/ConcAC0*100;
ConcB = b0;
ConcC0 = a0-ConcAC0;
ConcC=ConcC0;
ConcA0 = a0-ConcAC0-ConcAB0;
ConcA=ConcA0;

k1 = 0.002*60;
KD1 = ConcA0*ConcC0/ConcAC0;
k_1 = KD1*k1;
k2 = k1;
KD2 = KD1/50;
% may change for different analyte.
k_2 = KD2*k2;
k3 = 1*k1;
% change k3 from 0.001 to 10.
KD3 = KD2/KD1;
k_3 = KD3*k3;

for i = 1: (length(t)-1)
fab(i)= k2*ConcB(i)*ConcA(i) - k_2 *ConcAB(i) + k3 *ConcAC(i) * ConcB(i) - k_3*ConcAB(i)*ConcC(i);
fac(i)= k1*ConcC(i)*ConcA(i) - k_1 *ConcAC(i) - k3 *ConcAC(i) * ConcB(i) + k_3*ConcAB(i)*ConcC(i);
ConcAB(i+1) = ConcAB(i) + dt*fab(i);
ConcAC(i+1) = ConcAC(i) + dt*fac(i);
CD(i+1)=ConcAC(i+1)/ConcAC0*100;
ConcB(i+1) = ConcB(i);
ConcC(i+1) = a0-ConcAC(i+1);
ConcA(i+1) = a0-ConcAB(i+1)-ConcAC(i+1);
end

plot(t,CD, 'DisplayName','k3=k1', 'LineWidth',3)
% change k3 from 0.001k1 to 10k1.
xlabel('t (minutes)')
ylabel('CD (%)')
legend
```

```
lgd = legend;
lgd.FontSize = 18;
ax = gca;
ax.FontSize = 18;
ax.XLim = [0 120];
hold on
```

References:

(1) Sassanfar, M.; Szostak, J. W. An RNA Motif That Binds ATP. *Nature* **1993**, *364* (6437), 550–553. https://doi.org/10.1038/364550a0.

(2) Jiang, F.; Kumar, R. A.; Jones, R. A.; Patel, D. J. Structural Basis of RNA Folding and Recognition in an AMP–RNA Aptamer Complex. *Nature* **1996**, *382* (6587), 183–186. https://doi.org/10.1038/382183a0.

(3) Nakatsuka, N.; Yang, K.-A.; Abendroth, J. M.; Cheung, K. M.; Xu, X.; Yang, H.; Zhao, C.; Zhu, B.; Rim, Y. S.; Yang, Y.; Weiss, P. S.; Stojanović, M. N.; Andrews, A. M. Aptamer–Field-Effect Transistors Overcome Debye Length Limitations for Small-Molecule Sensing. *Science* **2018**, *362* (6412), 319–324. https://doi.org/10.1126/science.aao6750.

(4) Kuzyk, A.; Schreiber, R.; Zhang, H.; Govorov, A. O.; Liedl, T.; Liu, N. Reconfigurable 3D Plasmonic Metamolecules. *Nature Materials* **2014**, *13* (9), 862–866. https://doi.org/10.1038/nmat4031.

(5) Huang, Y.; Nguyen, M.-K.; Kuzyk, A. Assembly of Gold Nanorods into Chiral Plasmonic Metamolecules Using DNA Origami Templates. *JoVE* **2019**, No. 145, e59280. https://doi.org/10.3791/59280.

(6) Liu, B.; Liu, J. Freezing Directed Construction of Bio/Nano Interfaces: Reagentless Conjugation, Denser Spherical Nucleic Acids, and Better Nanoflares. *J. Am. Chem. Soc.* **2017**, *139* (28), 9471–9474. https://doi.org/10.1021/jacs.7b04885.

(7) Wang, R.; Zhang, Q.; Zhang, Y.; Shi, H.; Nguyen, K. T.; Zhou, X. Unconventional Split Aptamers Cleaved at Functionally Essential Sites Preserve Biorecognition Capability. *Anal. Chem.* **2019**, *91* (24), 15811–15817. https://doi.org/10.1021/acs.analchem.9b04115.

(8) Bottari, F.; Daems, E.; de Vries, A.-M.; Van Wielendaele, P.; Trashin, S.; Blust, R.; Sobott, F.; Madder, A.; Martins, J. C.; De Wael, K. Do Aptamers Always Bind? The Need for a Multifaceted Analytical Approach When Demonstrating Binding Affinity between Aptamer and Low Molecular Weight Compounds. *J. Am. Chem. Soc.* **2020**, *142* (46), 19622–19630. https://doi.org/10.1021/jacs.0c08691.

(9) McKeague, M.; De Girolamo, A.; Valenzano, S.; Pascale, M.; Ruscito, A.; Velu, R.; Frost, N. R.; Hill, K.; Smith, M.; McConnell, E. M.; DeRosa, M. C. Comprehensive Analytical Comparison of Strategies Used for Small Molecule Aptamer Evaluation. *Anal. Chem.* **2015**, *87* (17), 8608–8612. https://doi.org/10.1021/acs.analchem.5b02102.

(10) Cho, E. J.; Lee, J.-W.; Ellington, A. D. Applications of Aptamers as Sensors. *Annual Rev. Anal. Chem.* **2009**, *2* (1), 241–264. https://doi.org/10.1146/annurev.anchem.1.031207.112851.

(11) Nguyen, M.-K.; Kuzyk, A. Reconfigurable Chiral Plasmonics beyond Single Chiral Centers. *ACS Nano* **2019**, *13* (12), 13615–13619. https://doi.org/10.1021/acsnano.9b09179.

(12) Zadeh, J. N.; Steenberg, C. D.; Bois, J. S.; Wolfe, B. R.; Pierce, M. B.; Khan, A. R.; Dirks, R. M.; Pierce, N. A. NUPACK: Analysis and Design of Nucleic Acid Systems. *Journal of Computational Chemistry* **2011**, *32* (1), 170–173. https://doi.org/10.1002/jcc.21596.

(13) Chi, Q.; Wang, G.; Jiang, J. The Persistence Length and Length per Base of Single-Stranded DNA Obtained from Fluorescence Correlation Spectroscopy Measurements Using Mean Field Theory. *Physica A: Statistical Mechanics and its Applications* **2013**, *392* (5), 1072–1079. https://doi.org/10.1016/j.physa.2012.09.022.

(14) Roth, E.; Glick Azaria, A.; Girshevitz, O.; Bitler, A.; Garini, Y. Measuring the Conformation and Persistence Length of Single-Stranded DNA Using a DNA Origami Structure. *Nano Lett.* **2018**, *18* (11), 6703–6709. https://doi.org/10.1021/acs.nanolett.8b02093.

(15) Korpelainen, V.; Linko, V.; Seppä, J.; Lassila, A.; Kostiainen, M. A. DNA Origami Structures as Calibration Standards for Nanometrology. *Measurement Science and Technology* **2017**, *28* (3), 034001. https://doi.org/10.1088/1361-6501/28/3/034001.

(16) Huang, Y.; Nguyen, M.-K.; Nguyen, V. H.; Loo, J.; Lehtonen, A. J.; Kuzyk, A. Characterizing Aptamers with Reconfigurable Chiral Plasmonic Assemblies. *Langmuir* **2022**, *38* (9), 2954–2960. https://doi.org/10.1021/acs.langmuir.1c03434.
